# The Colitis-Associated Transcriptional Profile of Commensal *Bacteroides thetaiotaomicron* Enhances Adaptive Immune Responses to a Bacterial Antigen

**DOI:** 10.1371/journal.pone.0042645

**Published:** 2012-08-03

**Authors:** Jonathan J. Hansen, Yong Huang, Daniel A. Peterson, Laura Goeser, Ting-Jia Fan, Eugene B. Chang, R. Balfour Sartor

**Affiliations:** 1 Center for Gastrointestinal Biology and Disease, University of North Carolina at Chapel Hill, Chapel Hill, North Carolina, United States of America; 2 Department of Medicine, Division of Gastroenterology and Hepatology, University of North Carolina at Chapel Hill, Chapel Hill, North Carolina, United States of America; 3 Department of Medicine, Section of Gastroenterology, University of Chicago Medical Center, Chicago, Illinois, United States of America; 4 Department of Pathology, Johns Hopkins University School of Medicine, Baltimore, Maryland, United States of America; 5 Department of Microbiology and Immunology, University of North Carolina at Chapel Hill, Chapel Hill, North Carolina, United States of America; Massachusetts General Hospital, United States of America

## Abstract

**Background:**

Inflammatory bowel diseases (IBD) may be caused in part by aberrant immune responses to commensal intestinal microbes including the well-characterized anaerobic gut commensal *Bacteroides thetaiotaomicron* (*B. theta*). Healthy, germ-free HLA-B27 transgenic (Tg) rats develop chronic colitis when colonized with complex gut commensal bacteria whereas non-transgenic (nTg) rats remain disease-free. However, the role of *B. theta* in causing disease in Tg rats is unknown nor is much known about how gut microbes respond to host inflammation.

**Methods:**

Tg and nTg rats were monoassociated with a human isolate of *B. theta*. Colonic inflammation was assessed by histologic scoring and tissue pro-inflammatory cytokine measurement. Whole genome transcriptional profiling of *B. theta* recovered from ceca was performed using custom GeneChips and data analyzed using dChip, Significance Analysis of Microarrays, and Gene Set Enrichment Analysis (GSEA) software. Western Blots were used to determine adaptive immune responses to a differentially expressed *B. theta* gene.

**Results:**

*B. theta* monoassociated Tg rats, but not nTg or germ-free controls, developed chronic colitis. Transcriptional profiles of cecal *B. theta* were significantly different in Tg vs. nTg rats. GSEA revealed that genes in KEGG canonical pathways involved in bacterial growth and metabolism were downregulated in *B. theta* from Tg rats with colitis though luminal bacterial concentrations were unaffected. Bacterial genes in the Gene Ontology molecular function “receptor activity”, most of which encode nutrient binding proteins, were significantly upregulated in *B. theta* from Tg rats and include a SusC homolog that induces adaptive immune responses in Tg rats.

**Conclusions:**

*B. theta* induces colitis in HLA-B27 Tg rats, which is associated with regulation of bacterial genes in metabolic and nutrient binding pathways that may affect host immune responses. These studies of the host-microbial dialogue may lead to the identification of novel microbial targets for IBD therapies.

## Introduction

It is becoming increasingly clear that commensal intestinal bacteria provide functions that significantly impact not only normal host physiology, but also disease pathogenesis. For example, while certain symbiotic members of the human gut microbiome supply nutrients to the host, induce protective responses in the intestinal epithelium, and influence normal mucosal immune development, other members known as pathobionts have the capacity to induce disease in susceptible hosts or specific environmental conditions [1], [2]. The role of commensal bacteria in causing human disease is perhaps best illustrated by our current understanding of the pathogenesis of inflammatory bowel diseases (IBD).

IBD are a group of chronic intestinal inflammatory disorders, including Crohn's disease (CD) and ulcerative colitis, that are caused in part by dysregulated immune responses to commensal intestinal bacteria in genetically susceptible hosts. The role of host genetics in IBD is highlighted by the discovery of over 100 genes that are associated with IBD, more than 80 of which are linked to CD. NOD2, the CD susceptibility gene with the single largest effect size, and many of the other IBD susceptibility genes encode proteins that participate in host innate and adaptive immune responses to bacteria [3], [4]. Thus, genetic studies indicate that defective immune responses to bacteria may contribute to the development of IBD. The pathologic role of commensal bacteria in IBD is further substantiated by clinical studies in which surgical diversion of the fecal stream reduced inflammation in bypassed intestinal segments, suggesting that luminal contents contain pro-inflammatory components such as bacteria [5].

In addition to genetic and clinical studies, profiling the composition of the intestinal microbial community has also revealed associations between CD and commensal bacteria. In general, CD is associated with decreased bacterial diversity, increased numbers of Proteobacteria, and decreased numbers of Firmicutes [6]. Specifically, compared to healthy controls, increased numbers of functionally distinct commensal *Escherichia coli* belonging to the B2+D phylotypes and commensal adherent-invasive *E. coli* strains are present in the intestinal mucosa of patients with CD affecting the colon and ileum, respectively [7], [8]. Decreased numbers of the Firmicute, *Faecalibacterium prausnitzii*, are detected in intestinal mucosa from patients with post-operative recurrence of ileal CD compared to non-inflamed controls [9]. In contrast to Proteobacteria and Firmicutes, changes in the number of commensals belonging to the Bacteroidetes, one of the dominant phyla in the human gut microbiome, are variable in patients with IBD [10], [11], [12], [13], [14]. However, host immune responses to a member of the Bacteroidetes, *Bacteroides thetaiotaomicron* (*B. theta*), are elevated in colonic tissue from CD patients [15]. Together, these studies suggest that changes in the composition of the gut microbial community and aberrant immune responses to Bacteroidetes are associated with human IBD.

Studies of animal models have also provided remarkable insights into the pathogenesis of IBD. Similar to results from human studies, Proteobacteria are consistently expanded in animal models of IBD, whereas changes in Bacteroidetes are variable depending upon the model and method of detection [6]. The most direct evidence linking commensal bacteria to the development of IBD comes from gnotobiotic animal studies, in which virtually all animal models of IBD that have been tested are healthy in the germ-free (GF) state, but develop intestinal inflammation when exposed to viable commensal bacteria [16]. For example, Rag1-/- mice that are selectively colonized (monoassociated) with a human isolate of *B. theta* have increased innate immune responses in the small intestine compared to GF mice [17]. Treatment with antibiotics that eliminate certain members of the commensal microbiota abrogates spontaneous colitis in Il10R2/TGFBR2 double knockout mice, but colitis recurs when these mice are inoculated with murine isolates of *Bacteroides* spp., especially *B. theta* and *Bacteroides vulgatus*, but not *E. coli* [18]. In another model, transgenic rats harboring the human *HLA-B27* and *β2 microglobulin* genes (Tg) remain healthy when housed in germ-free conditions, but develop spontaneous multi-organ inflammation, including colitis, when colonized with commensal bacteria [19], [20]. Furthermore, gnotobiotic experiments in which germ-free *HLA-B27* Tg rats are selectively colonized with defined bacteria revealed that not all commensal bacteria have equal colitogenic potential. For example, *B. vulgatus* causes worse inflammation than *B. distasonis* and *E. coli* causes no inflammation in *HLA-B27* Tg rats monoassociated with specific strains of these bacteria [20], [21].

While others have shown that *B. theta* may contribute to the pathogenesis of human IBD and experimental murine colitis, relatively little is known about its role in other models of intestinal inflammation, nor is much known about how host inflammation affects *B. theta* function. Herein, we present data that a human isolate of commensal *B. theta* causes chronic colitis in monoassociated *HLA-B27* Tg rats, which in turn induces transcriptional changes in luminal bacterial genes that impact host immune responses.

## Materials and Methods

### Bacterial Cultures

The fully-sequenced human fecal isolate of *B. theta* (VPI-5482) was grown on Brain-Heart Infusion (BHI) agar and in BHI broth under strict anaerobic conditions using pre-reduced media. To quantify viable luminal bacteria, weighed, frozen cecal contents were thawed and diluted in PBS in an anaerobic chamber and grown on BHI agar for 3 days. For dilution calculations, we assumed that 1 g of contents has a volume of 1 mL. Results were expressed as colony forming units (CFU)/g of contents.

### Animal Experiments

Four adult germ-free HLA-B27/β2 microglobulin Tg rats and five adult germ-free nTg littermate controls on the Fisher F344 background [19] were monoassociated for six weeks in gnotobiotic isolators at the National Gnotobiotic Rodent Resource Center at UNC Chapel Hill. At necropsy, serum was collected, luminal contents and intestinal tissue were snap frozen in liquid nitrogen for RNA isolation, and intestinal tissue was fixed in formalin for histologic processing. Contamination of animals with other bacteria was excluded by Gram staining and plating of cecal contents on BHI agar, which was cultured aerobically and anaerobically for seven days.

### Ethics Statement

Animals were treated humanely throughout the entire experiment. All animal protocols were approved by the Institutional Animal Care and Use Committee at the University of North Carolina at Chapel Hill (Protocol 09-239.0).

### Histologic Scoring

Formalin-fixed, paraffin-embedded intestinal tissue was stained with hematoxylin and eosin and inflammation was quantified using a validated blinded histological scoring system as described previously [22].

### RNA Isolation

Mammalian RNA was isolated from intestinal tissue using Trizol Reagent (Invitrogen, Carlsbad, CA) according to the manufacturer's instructions. Bacterial RNA was isolated from rat cecal contents as previously described [17].

### Microarray Analysis

Bacterial cDNA was synthesized and hybridized to 8 custom GeneChips (1 GeneChip per animal, 4 animals per group) as previously described [17]. The chip quality check and data normalization were processed using dChip software (http://biosun1.harvard.edu/complab/dchip/). Unsupervised hierarchical clustering of samples was also carried out by dChip software. Briefly, 6 arrays passed the established criteria for sample and chip outlier identification. We used “PM-only” background correction, “invariant set” normalization and model-based summary of gene expression levels [23]. The .cel files and normalized data were deposited in NCBI GEO database with accession number GSE34966.

We filtered the genes using criterion of coefficient of variation >0.5 and <2, resulting in 180 *B. theta* genes demonstrating large variation across all samples. Unsupervised hierarchical clustering of samples was performed based on the expression profile of these 180 genes using dChip software with “1-Pearson correlation” for distance metric and “centroid” for linkage. Three-dimensional principal components analysis was performed on normalized microarray data from the entire GeneChip using Genespring GX 11.0 software with “pruning” set at 4 eigenvectors, mean centered normalization, and scale to unit standard deviation.

Differentially expressed genes between nTg and Tg animals were identified by two-group comparison using Significance Analysis of Microarrays [24]. The criteria for identification of differentially expressed genes were fold change >1.5 and false discovery rate (FDR)<4%.

### Pathways Analysis of Transcriptional Profiles

The KEGG pathways of *B. theta* were curated from NCBI BioProject Genome Project Accession No. 62913 and RefSeq NC_004663.1[25]. The *B. theta* KEGG database contains 84 metabolism and signaling pathways including 860 genes, 89% of which were covered by the approximately 5000 *B. theta* genes on the Affymetrix Human gut microbiota community GeneChip used in these experiments. The metabolism and signaling pathways significantly distinct between two experiment groups were identified using Gene Set Enrichment Analysis (GSEA) software (http://www.broadinstitute.org/gsea/index.jsp). GSEA is a computational method that determines whether an a priori defined set of genes shows statistically significant, concordant differences between two biological states [26]. Briefly, the normalized microarray data was converted into .GCT format, while the chip annotation file was converted into .CHIP format. The canonical pathways of *B. theta* were downloaded from the KEGG database (http://www.genome.jp/kegg/) and converted into .GMT format, as required by GSEA submission. The criterion for significant pathways was set at FDR<20%.

GSEA was also used to perform Gene Ontology (GO) analysis similar to KEGG analysis. GO annotation of the *B. theta* genome was performed as originally described [25], [27] and was updated in the Gordon lab in 2008, http://gordonlab.wustl.edu/sonnenburg/index.html (unpublished).

### Quantitative Real-time RT-PCR

Cytokine and bacterial transcripts were quantified by real-time RT-PCR using the ΔΔCt relative quantification method as described previously [28]. Rat β-actin and *B. theta* 16S were chosen as reference genes. Primer pairs are listed in Table S3. Mammalian primers spanned introns.

### Sequence Alignment, Cloning and Purification of BT4357 Fragment

Amino acid sequence alignment between BT4357 and *Bacteroides caccae* OmpW was performed using web-based ClustalW2 software (http://www.ebi.ac.uk/Tools/msa/clustalw2/). A fragment of BT4357 corresponding to amino acid residues 160 to 741 was amplified from *B. theta* genomic DNA using PCR primers 5-attccatatgaaaggctccgccaatggtacga-3′ and 5′-cgcggatccttatacagaagggaagaaaccgaaac-3′ and subcloned into the PET15b expression vector (EMD Millipore, Billerica, MA) using standard molecular biology procedures. The cloned portion of BT4357 excludes the predicted leader peptide and a portion of the transmembrane domain in order to facilitate overexpression and purification, but includes the region that correlates to the putative epitope in *B. caccae* OmpW. The BT4357 protein fragment was overexpressed in *E. coli* BL21-DE3 (EMD Millipore, Billerica, MA) and purified using nickel agarose (Qiagen, Valencia, CA) according to the manufacturer's instructions.

### Western Blots

Ten mcg of purified BT4357 fragment were separated on 10% SDS-polyacrylamide gels and transferred to nitrocellulose membranes using standard protocols. Membranes were stained with Ponceau S for five minutes and then rinsed with water to visualize protein bands. Individual membrane strips from each lane were incubated in 5% non-fat dry milk in TBST (TBST-milk) for 60 min, 1∶300 rat sera in TBST-milk for 90 min, 1∶4000 goat anti-rat IgG-HRP (SantaCruz Biotechnology, Santa Cruz, CA) in TBST-milk for 60 min, and ECL Prime reagent (Amersham, Piscataway, NJ) for five minutes. Immunoreactive bands were detected by exposing strips to X-ray film.

## Results

### Human Commensal *B. theta* Induces Chronic T-cell-mediated Inflammation in *HLA-B27* Tg Rats

**Figure 1:**
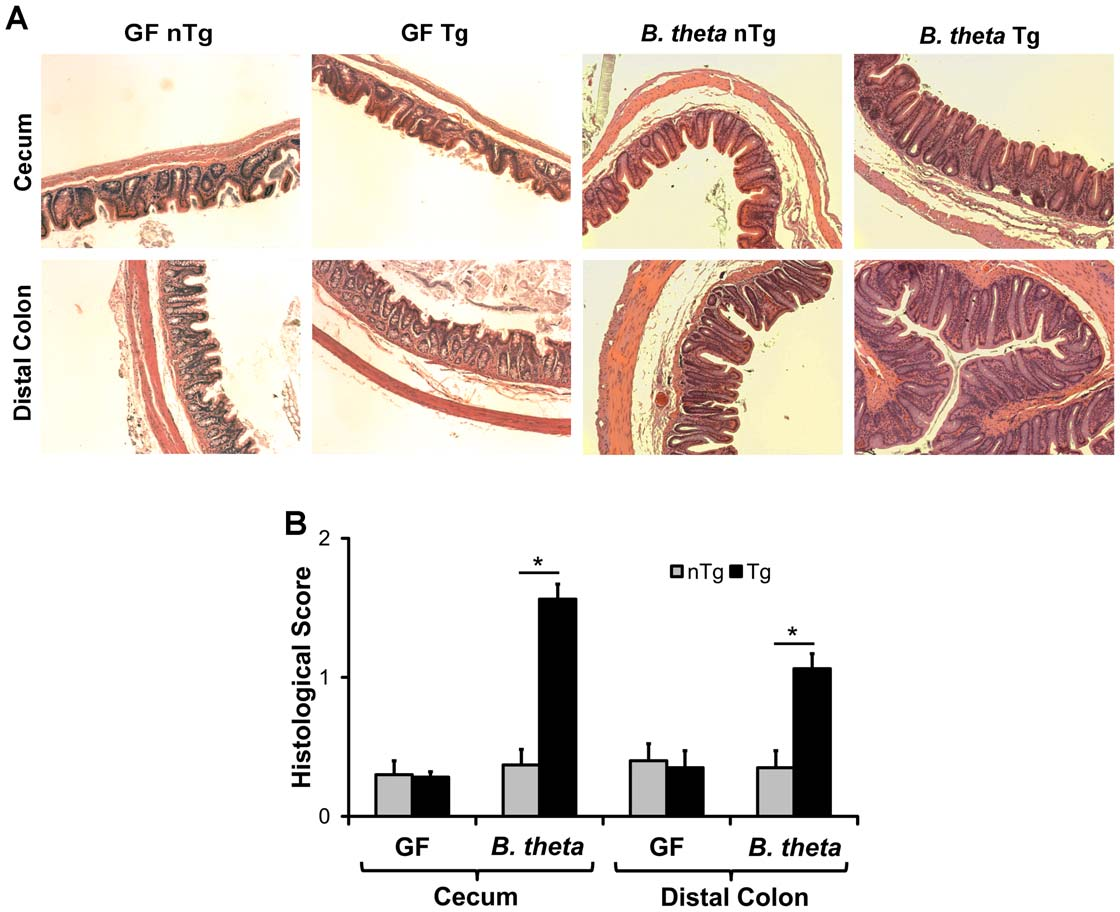
Colonic Inflammation in Germ-Free and *B. theta* Monoassociated Rats. (A) Representative photomicrographs of H&E stained cecum and distal colon pieces from germ-free (GF) and 6-week *B. theta*-monoassociated non-transgenic (nTg) and *HLA-B27* transgenic (Tg) rats taken using a 10× objective. (B) Blinded histological inflammation scores of cecum and distal colon tissue from GF and *B. theta*-monoassociated rats (mean+SEM, *p<0.05, n = 4–5 rats/group).

Since others have previously shown that selective colonization of *HLA-B27* Tg rats with *B. vulgatus* induces variable degrees of chronic intestinal inflammation and since *B. theta* has been associated with human IBD and a model of murine colitis, we wanted to determine whether *B. theta* causes colitis in monoassociated Tg rats similar to that observed in human IBD. We therefore colonized germ-free Tg and non-transgenic (nTg) rats with the human commensal isolate of *B. theta* (VPI-5482) for 6 weeks and measured histological inflammation. We found significantly greater inflammation in the ceca and distal colons of Tg compared to nTg and germ-free rats, which was characterized primarily by increased lamina propria mononuclear cell infiltrate and mild crypt hyperplasia (Figure 1).

**Figure 2:**
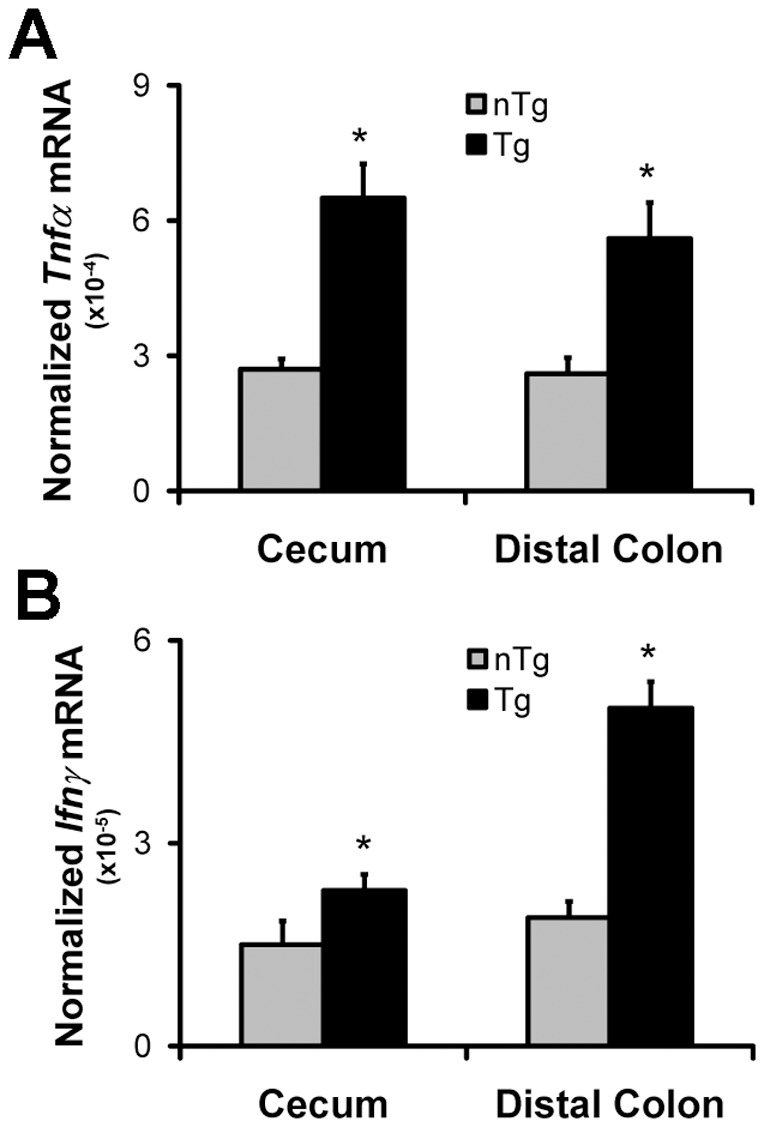
Expression of Pro-Inflammatory Cytokines in Colon Tissue from *B. theta* Monoassociated Rats. Abundance of TNFα (A) and IFNγ (B) mRNA normalized to β-actin as measured by real-time RT-PCR in cecum and distal colon tissue from 6-week monoassociated non-transgenic (nTg) and *HLA-B27* transgenic (Tg) rats (mean+SEM, *p<0.05, n = 4 rats/group).

To determine whether the histological inflammation was associated with enhanced T-cell-mediated cytokine expression in colonic tissue from Tg rats, we performed real-time PCR of selected cytokines and found that TNFα and IFNγ transcripts were significantly higher in cecal and distal colon tissue from monoassociated Tg compared to nTg rats (Figure 2). Together, these data indicate that a human isolate of *B. theta* induces colitis in a Tg rat model of IBD and that the inflammation is characterized by upregulation of pro-inflammatory cytokines that mediate T-cell responses.

### Intestinal Inflammation is Associated with Alterations in the *B. theta* Transcriptome

While much is known about the effects of commensal bacteria on host immune responses, relatively little is known about how host inflammation affects luminal bacterial function. It is plausible that chronic intestinal inflammation generates an environment that alters the function or colitogenic potential of commensal bacteria. We have previously reported that stress response genes are upregulated and tricarboxylic acid cycle genes are downregulated in commensal *E. coli* from monoassociated IL-10-/- mice with overt histologic colitis compared to *E. coli* from healthy wild-type mice [28]. Furthermore, two genes that are upregulated in luminal *E. coli* during experimental colitis may negatively impact survival and inflammatory potential of the bacteria in genetically susceptible hosts [28]. We have also shown in *B. theta* monoassociated Rag1-/- mice, which have molecular evidence of increased innate immune responses, that luminal bacteria upregulate pathways that metabolize host innate immune effector molecules [17].

**Figure 3:**
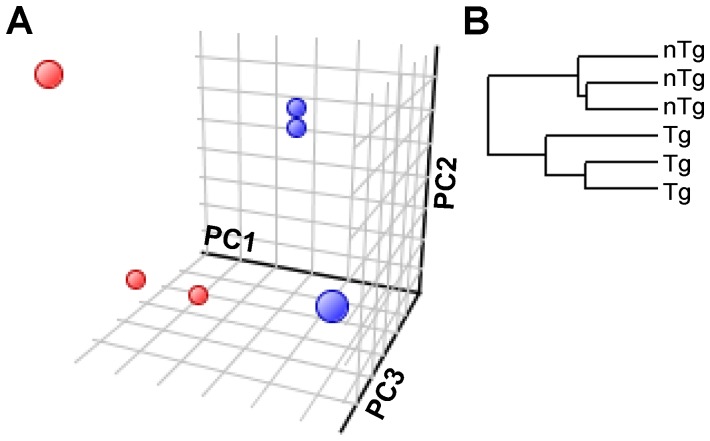
Transcriptional Profiles of *B. theta* Recovered from the Ceca of Monoassociated Rats. (A) 3-dimensional principal components analysis and (B) unsupervised hierarchical clustering of transcriptomes of cecal *B. theta* from 6-week monoassociated rats (PC1 = 36.1%, PC2 = 23.9%, PC3 = 21.7%). Red and blue spheres represent bacterial transcriptomes from *HLA-B27* transgenic (Tg) and non-transgenic (nTg) rats, respectively.

However, whether chronic, T-cell mediated inflammation affects *B. theta* gene expression is unknown. We therefore performed whole-genome transcriptional profiling with custom *B. theta* GeneChips containing probe sets that cover 98.6% of the bacterium's 4779 protein-coding genes [17], [27]. Using principle component analysis and unsupervised hierarchical clustering, we found that the *B. theta* transcriptome was significantly different in cecal bacteria from Tg rats with inflammation compared to healthy nTg (Figure 3A and 3B). We detected 329 differentially expressed transcripts representing 44 Kyoto Encyclopedia of Genes and Genomes (KEGG) canonical pathways. These findings indicate that host genotype (i.e. inflammation) significantly impacts luminal bacterial gene expression in this model.

To identify the individual *B. theta* genes that are significantly affected by inflammation, we performed two-group comparison using Significance Analysis of Microarrays and a fold-change threshold of 1.5 and false discovery rate of <0.05 [24]. We identified 56 genes that were upregulated and 273 genes that were downregulated in *B. theta* from Tg rats compared to nTg rats (Tables S1 & S2). Most of the highly upregulated genes encode membrane proteins of the starch utilization system (Sus) in *B. theta*. BT_3969, the second most highly upregulated gene, encodes a cation efflux protein of the Acr family that may be involved in exporting potentially toxic molecules including bile salts, indole, and antimicrobial peptides. A larger set of genes were downregulated in bacteria from Tg rats. However, many of the most highly downregulated genes encode hypothetical proteins for which there is no known function. Of the most highly downregulated genes with known or predicted functions, many encode proteins that are involved in cell wall biosynthesis and ribosome assembly. Real-time RT-PCR confirmed microarray results of several selected genes in multiple metabolic pathways (Figure S1). These data suggest that *B. theta* responds to inflammation by upregulating genes that facilitate nutrient uptake and survival and downregulating genes that are involved in growth and metabolism.

### *B. theta* downregulate metabolic pathways during experimental colitis

Since differential expression of individual genes does not necessarily correlate with entire pathways that are significantly affected by an experimental condition, we performed pathways analysis to test our hypothesis that *B. theta* downregulates processes of growth and cell division and upregulates processes that facilitate persistence and nutrient acquisition. We used Gene Set Enrichment Analysis (GSEA) software to analyze gene ontology (GO) molecular functions and KEGG canonical pathways that were significantly changed in *B. theta* from Tg compared to nTg rats. GSEA is a computational method that determines whether an *a priori* defined set of genes shows statistically significant, concordant differences between two biological states.

**Figure 4:**
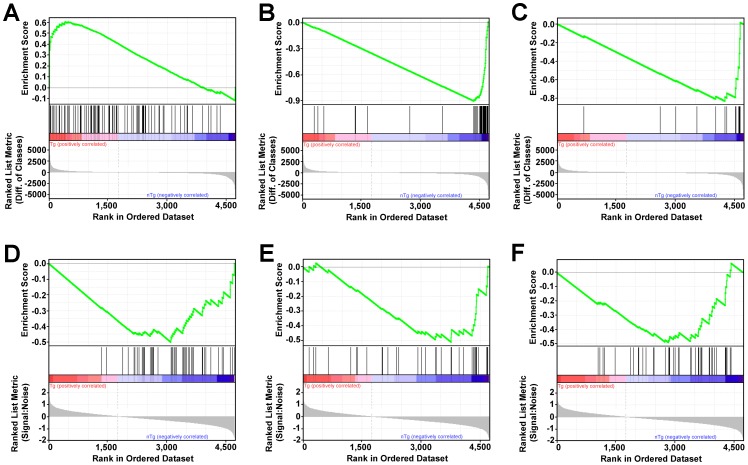
GSEA Enrichment Plots of GO Molecular Functions and KEGG Canonical Pathways With the Largest Normalized Enrichment Scores. (A) Receptor Activity (GO0004872), (B) Structural Constituent of Ribosome (GO0003735), (C) Hydrogen Ion Transporting ATPase Activity (GO0046961), (D) Ribosome, (E) Oxidative Phosphorylation, (F) Pyrimidine Metabolism. Top portions of the plots show the running enrichment score for the gene set as the analysis “walks down” the ranked list. Middle portions of the plots shows where members, indicated by vertical black lines, of the gene set appear in the ranked list of genes. Bottom portions of the plots show the value of the ranking metric as one progresses down the list of ranked genes.

Virtually all of the significantly affected *B. theta* pathways (FDR q<0.20) were enriched with genes that were downregulated in *B. theta* from Tg rats with colitis compared to *B. theta* from healthy nTg rats (Tables 1 and 2). GO molecular functions involved in translation and ATP production, including “Structural Constituent of Ribosome” and “Hydrogen Ion Transporting ATPase Activity,” were significantly enriched with genes that are downregulated in cecal bacteria from Tg rats (Table 1, Figure 4B–C). Similar to the results of the GSEA analysis of GO molecular functions, GSEA analysis of KEGG canonical pathways revealed that “Ribosome” and “Oxidative Phosphorylation” were highly enriched with genes that are downregulated in cecal *B. theta* from Tg rats (Table 2, Figure 4D–E). In addition, “Pyrimidine Metabolism,” “Purine Metabolism”, “Peptidoglycan Biosynthesis”, and “Metabolic Pathways” were significantly enriched with *B. theta* genes that are downregulated in Tg rats with inflammation (Table 2, Figure 4F). These results indicate that colitis is predominantly associated with downregulation of genes in *B. theta* pathways important for energy production, metabolism, and growth.

**Table 1:** GO Molecular Functions that are Enriched with Genes Downregulated in *B. theta* from Monoassociated HLA-B27 Tg Rats.

GO Molecular Function	Number of Genes in Pathway	Normalized Enrichment Score	FDR q value
Structural Constituent of Ribosome (GO0003735)	46	−2.01	<0.001
Hydrogen Ion Transporting ATPase Activity (GO0046961)	13	−1.55	0.094
Hydrogen Ion Transporting ATP Synthase Activity (GO0046933)	13	−1.52	0.110
Cation Binding (GO0043169)	54	−1.50	0.101
Protein Binding (GO0005515)	15	−1.50	0.085
Binding (GO0005488)	40	−1.48	0.094
Hydrolase Activity, Acting on Acid Anhydrides, Catalyzing Transmembrane Movement of Substances (GO0016820)	10	−1.44	0.145
Electron Carrier Activity (GO0009055)	36	−1.44	0.130
Cation Transporter Activity (GO0008324)	11	−1.39	0.189
RNA Binding (GO0003723)	38	−1.35	0.257

**Table 2:** KEGG Canonical Pathways that are Enriched with Genes Downregulated in *B. theta* from Monoassociated HLA-B27 Tg Rats.

KEGG Pathway	Number of Genes in Pathway	Normalized Enrichment Score	FDR q value
Ribosome	51	−1.77	0.048
Oxidative Phosphorylation	35	−1.66	0.098
Pyrimidine Metabolism	42	−1.63	0.081
Purine Metabolism	49	−1.51	0.197
Peptidoglycan Biosynthesis	16	−1.49	0.184
Fructose and Mannose Metabolism	27	−1.44	0.221
Metabolic Pathways	478	−1.44	0.191
Pyruvate Metabolism	25	−1.42	0.195
Aminoacyl-tRNA Biosynthesis	21	−1.41	0.189
Pentose Phosphate Pathway	22	−1.35	0.245

### Luminal *B. theta* viability is preserved during colitis despite down regulation of cell division genes

**Figure 5:**
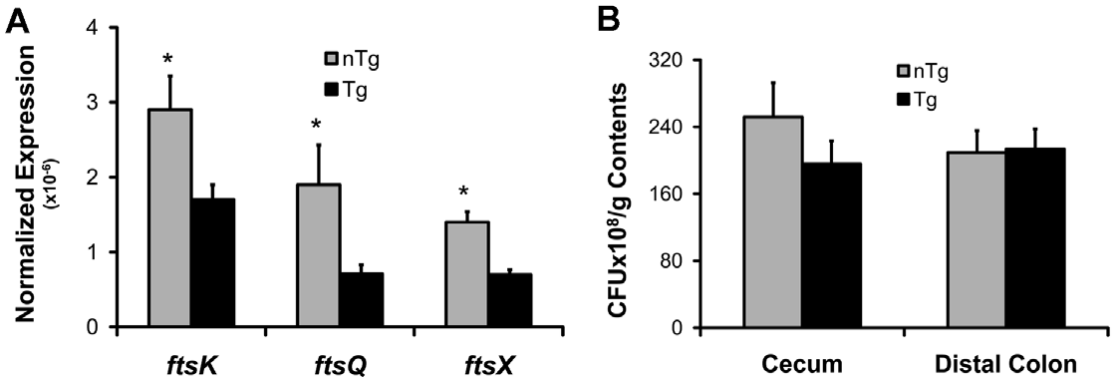
Expression of Cell-Division Genes and Bacterial Density in Cecal Contents from *B. theta* Monoassociated Rats. (A) Normalized expression of *fts* cell-division genes relative to bacterial 16S rRNA in *B. theta* from ceca of 6-week monoassociated rats. (B) Colony forming units (CFU)/g cecal contents from 6-week monoassociated non-transgenic (nTg) and *HLA-B27* transgenic (Tg) rats (mean+SD, n = 4–5 rats/group, *p<0.05).

We next wanted to confirm the results of the pathways analysis by measuring molecular markers of bacterial cell division and quantifying luminal bacterial concentrations. To indirectly test whether cell division was decreased in luminal bacterial from Tg rats with colitis, we measured expression of *fts* genes, which are critical for bacterial septum formation and cell division [29]. We detected significantly fewer transcripts of *ftsK*, *ftsQ*, and *ftsX* in cecal bacteria from Tg compared to nTg rats (Figure 5A). Based on the pathways analysis and qPCR results of *fts* genes, we hypothesized that luminal bacterial numbers would also be decreased in Tg compared to nTg rats. However, while we detected numerically fewer viable bacteria per gram of cecal contents in Tg vs. nTg rats, the difference was not statistically significant (Figure 5B). Moreover, there was no difference in bacterial density in the distal colon of Tg rats compared to nTg rats. Thus, downregulation of genes in metabolic pathways associated with bacterial cell growth and division in cecal *B. theta* from Tg rats did not correlate with a statistically significant decreased density of viable luminal bacteria. These data suggest that in the inflamed intestine, *B. theta* may compensate for downregulated pathways important in bacterial cell growth and division by upregulating other pathways that promote bacterial persistence and maintain viability.

### Luminal *B. theta* from rats with colitis upregulate genes with receptor activity

In the pathways analysis we detected only one feature, GO molecular function “Receptor Activity,” that was significantly enriched (FDR q<0.2) with genes that are upregulated in *B. theta* from Tg rats with colitis (Table 3, Figure 4A). No KEGG canonical pathways were significantly enriched with genes that are upregulated in cecal *B. theta* from Tg rats (Table 4). Interestingly, 98% of the *B. theta* genes assigned to the GO category “Receptor Activity” encode TonB-dependent receptors probably involved in binding nutrients including iron-siderophore complexes, vitamin B12, and glycans [30], [31]. When we examined the list of genes that were most highly upregulated in *B. theta* from Tg rats with colitis, we found that 3 out of the top 5 upregulated *B. theta* genes (BT2259, BT2260, and BT4357) encode TonB-dependent receptors (Table S1). Therefore, we speculate that *B. theta* in rats with colitis may upregulate TonB-dependent receptors to improve nutrient acquisition and viability.

**Table 3:** GO Molecular Functions that are Enriched with Genes Upregulated in *B. theta* from Monoassociated HLA-B27 Tg Rats.

GO Molecular Function	Number of Genes in Pathway	Normalized Enrichment Score	FDR q value
Receptor Activity (GO0004872)	93	1.70	0.148
Transporter Activity (GO0005215)	169	1.45	0.742
Sigma Factor Activity (GO0016987)	37	1.29	1.000
Transferase Activity, Transferring Nitrogenous Groups (GO0016769)	10	1.28	0.875
Metallopeptidase Activity (GO0008237)	16	1.27	0.713
Transcription Factor Activity (GO0003700)	140	1.26	0.625
Two-component Sensor Activity (GO0000155)	64	1.12	0.946
Calcium Ion Binding (GO0005509)	26	1.10	0.889
Sequence-Specific DNA Binding (GO0043565)	87	1.06	0.891
Protein Histidine Kinase Activity (GO0004673)	57	1.03	0.896

**Table 4:** KEGG Canonical Pathways that are Enriched with Genes Upregulated in *B. theta* from Monoassociated HLA-B27 Tg Rats.

KEGG Pathway	Number of Genes in Pathway	Normalized Enrichment Score	FDR q value
Nitrogen Metabolism	21	1.29	0.774
Two-component System	15	1.16	0.698
Sphingolipid Metabolism	15	0.87	1.000
Starch and Sucrose Metabolism	28	0.87	1.000
Other Glycan Degradation	24	0.79	0.964
Glycine, Serine, and Threonine Metabolism	20	0.72	0.890

### Transgenic rats develop adaptive immune responses to a *B. theta* TonB-dependent receptor

**Figure 6:**
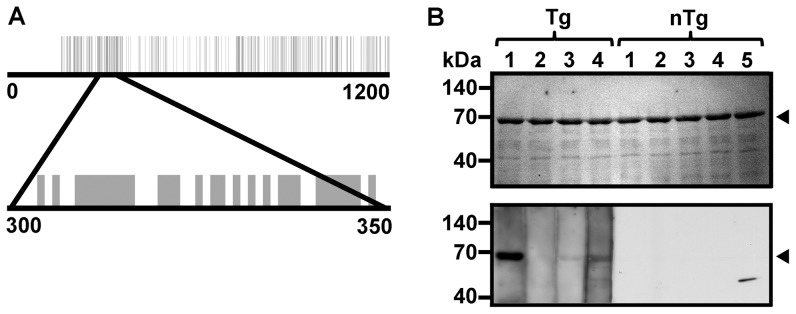
Serologic Responses to Recombinant BT4357 SusC Homolog in *B. theta* Monoassociated Rats. (A) Amino acid sequence alignment of BT4357 and *B. caccae* OmpW using Clustal W2, including a magnified view from residues 300–350. Vertical lines represent identical residues. (B) Ponceau S stained Western Blot membrane demonstrating equivalent amounts of purified recombinant BT4357 fragment in each lane (top panel). Western Blot of strips of the membrane shown in the top panel using sera from individual *B. theta* monoassociated transgenic (Tg) and non-transgenic (nTg) rats and an anti-rat IgG secondary antibody (bottom panel). Predicted size of BT4357 fragment is indicated by the arrowheads.

Because we found that expression of TonB-dependent receptors is increased in *B. theta* from rats with colitis, we searched the literature for an association between TonB-dependent receptors and human IBD. Intriguingly, others have previously reported that pediatric IBD patients have increased serum antibody titers against a TonB-dependent receptor from human commensal *Bacteroides caccae* named OmpW. Using sequence alignment, we determined that OmpW shares 27% overall amino acid similarity to one of the most highly upregulated TonB-dependent receptors in *B. theta* from Tg rats, BT4357 (Figure 6A) [32], [33]. Moreover, amino acid residues 300 to 350 in BT4357 are 58% similar to residues in the corresponding epitope-containing portion of *B. caccae* OmpW [32] (Figure 6A). To determine whether BT4357 is antigenic in Tg rats with colitis, we performed Western Blots using individual sera from *B. theta* monoassociated Tg and nTg rats to detect recombinant protein expressed from a cloned segment of BT4357 (amino acid residues 160–741). We detected BT4357-reactive IgG antibodies in sera from three of four *B. theta* monoassociated Tg rats, but none of five nTg rats (Figure 6B, lower panel). The elevated background signal in Tg samples may be due to increased levels of total serum IgG in these rats. Based on these data, we propose that chronic colitis causes luminal *B. theta* to upregulate the TonB-dependent receptor BT4357, which is antigenic in Tg, but not nTg rats. Whether BT4357 plays a direct role in the pathogenesis of IBD in this animal model remains to be established.

## Discussion

*B. theta* has been implicated in the pathogenesis of human IBD and a model of murine colitis. Here, we show that *B. theta* colonization is also sufficient to induce colitis in *HLA-B27* Tg rats. Furthermore, we demonstrate that chronic intestinal inflammation is associated with transcriptional changes in luminal *B. theta* that suggest decreased bacterial metabolism, growth, and division, but increased nutrient acquisition with the net effect of preserved bacterial viability. We also provide evidence that chronic colitis induces *B. theta* to upregulate expression of a gene that stimulates adaptive immune responses in genetically susceptible animals. Together, these findings indicate that commensal *B. theta* may adapt to intestinal inflammation by shifting cellular resources that would otherwise support cell division to processes that promote microbial persistence, and at the same time activate host immune responses.

The notion that bacteria respond to environmental stimuli by enhancing survival at the expense of growth has been documented elsewhere. For example, a commensal murine isolate of *E. coli* upregulates stress response genes in the IL-10-/- mouse model of colitis that decrease luminal bacterial densities *in vivo*, but have no effect on growth or persistence *in vitro* [28]. Additionally, a survey of many *E. coli* isolates has shown that high levels of σ^s^, a transcription factor that controls expression of stress-response genes, correlates with increased resistance to environmental stress, but decreased ability to metabolize low concentrations of nutrients [34]. Thus, the functional outcome of bacterial responses to environmental conditions can lead to growth or persistence depending on the nature of the stimulus and the genetic composition of the bacteria.

Whether the transcriptional changes noted in *B. theta* monoassociated Tg rats are representative of Bacteroides gene expression in human IBD remains to be determined. The animal model used in our studies has several limitations. While *Bacteroides spp*. are a dominant member of the commensal human gut microbiota, *B. theta* represents a relatively small component of the *Bacteroides* and changes in its transcriptome may not be representative of transcriptional changes that occur in other *Bacteroides spp*. Inflammation in *HLA-B27* Tg rats colonized with commensal bacteria is similar to that observed in human Crohn's disease because it is characterized by a Th-1 predominant immune response and affects both the upper and lower digestive tract and extraintestinal organs [19], [20]. However, unlike human Crohn's disease, Tg rats do not typically develop mucosal ulceration. It is also important to note that *B. theta* transcriptional changes in monoassociated rats used in our studies may not reflect those observed in a more natural complex microbiota where other members of the microbial community would likely influence *B. theta* gene regulation. Furthermore, our gene expression studies did not distinguish between luminal and mucosal-associated bacteria, which may be more relevant in host-commensal interactions due to proximity. However, we have previously shown that metagenomic functional profiles of mucosal associated bacteria are similarly diverse as those reported in feces [35].

Interestingly, most of the differentially expressed *B. theta* genes were downregulated in bacteria from Tg rats. Concordantly, more pathways were significantly enriched with genes that were downregulated in bacteria from Tg rats compared to pathways enriched with genes that were upregulated in bacteria from Tg rats. However, the transcriptional changes did not significantly affect luminal bacterial density and it is unknown whether deletion of differentially expressed genes in *B. theta* would affect bacterial viability in animals with colitis. While we have assumed that decreased expression of genes in metabolic and growth-related pathways should result in fewer viable luminal bacteria, it is possible that downregulation of these genes in bacteria does not affect growth. Consistent with this, differentially expressed genes in *B. theta* from the Tg rats did not overlap with genes in *B. theta* from wild-type mice, that when mutated, conferred a competitive growth advantage or disadvantage [36].

Compared to the number of downregulated genes, fewer bacterial genes were upregulated in Tg rats. For instance, *B. theta* upregulate expression of bacterial enzymes that metabolize products of the host innate immune response, including reactive oxygen and nitrogen species that are increased in intestinal tissue from patients with IBD and animals with experimental colitis [37], [38]. We found that bacteria from Tg rats with colitis increase expression of genes encoding nitrite reductase (BT1414–1417), an enzyme that detoxifies nitric oxides produced by activated host innate immune cells [39], compared to healthy controls. Expression of this same set of genes was increased in *B. theta* from monoassociated Rag1-/- mice that have increased levels of intestinal inducible nitric oxide synthase [17].

Many of the other highly upregulated genes encode proteins important for starch utilization by *B. theta*, including several s*usC* and *susD* homologues. Similarly, GO molecular function analysis revealed an enrichment in transcripts of genes with receptor activity in bacteria from Tg rats. *B. theta* have evolved mechanisms to utilize carbohydrates from a variety of sources—including dietary and host glycans [27], [40]. Genes involved in host mucin-O-glycan metabolism (BT1042–1053, BT4355–4359, and BT0317–0319), which were previously shown to enhance fitness of *B. theta in vivo* and participate in the production of the polysaccharide capsule, are upregulated in bacteria from Tg rats [31], [41]. This suggests that colitis is associated either with an altered carbohydrate landscape on the intestinal epithelium or decreased concentrations of dietary glycans in the cecum.

Interestingly, the *B. theta* s*usC* homolog (BT4357) that was most highly upregulated in Tg rats is very similar to *Bacteroides caccae* OmpW, which induces adaptive immune responses in pediatric IBD patients more frequently than in healthy controls [32], [33]. Both BT4357 and OmpW are TonB-dependent receptors and belong to a family of receptors that facilitate nutrient acquisition and uptake. In our studies, we found that BT4357 is also antigenic in Tg, but not nTg monoassociated rats suggesting the possibility of a conserved epitope in TonB-dependent receptors across *Bacteroides* species. However, it should be noted that we measured anti-BT4357 IgG in the rat sera, whereas the anti-OmpW antibodies detected in IBD patients were of the IgA isotype. Furthermore, it is possible that the elevated serologic immune responses to BT4357 in Tg rats are due to increased gut permeability during colitis and are not proportional to the expression level of BT4357 in luminal bacteria. While several other bacterial proteins including OmpC, I2 and flagellin have been previously shown to induce elevated antibody responses in IBD patients compared to controls, this is the first report of inflammation-associated upregulation of a bacterial antigen in colitis [42], [43]. We speculate that environmental factors in the lumen of the inflamed colon (e.g. altered nutrient availability) cause *B. theta* to induce expression of this TonB-dependent receptor, which is a dominant antigen that may contribute to chronic inflammation. Gnotobiotic studies using BT4357-deficient *B. theta* in Tg rats are needed to test whether immune responses to BT4357 are a cause or effect of colitis. Furthermore, testing sera from IBD patients for anti-BT4357 antibodies could expand the relevance of our findings to human IBD.

In summary, we have observed that the human commensal *B. theta* induces chronic, immune-mediated colitis in HLA-B27 Tg rats, which is associated with significant changes in luminal bacterial transcription that affect metabolic, growth, and nutrient binding pathways. In addition, intestinal inflammation induces *B. theta* to upregulate expression of a nutrient receptor that stimulates adaptive immune responses specifically in rats that are genetically susceptible to colitis. These studies add to a growing body of evidence demonstrating that commensal intestinal microbes actively participate in chronic intestinal inflammation by inducing and reacting to host inflammation. Exploring mechanisms of how host inflammatory factors affect gut commensals and the impact of inflammation-induced microbial transcriptional changes on disease pathogenesis may reveal novel pathways that could be potential therapeutic targets to treat human IBD.

## Supporting Information

Figure S1 **Real-Time RT-PCR Verification of Microarray Results for Selected** ***B. theta*** **Genes.** Selected differentially expressed *B. theta* genes from cecal bacteria from 6 week-monoassociated non-transgenic (nTg) and *HLA-B27* transgenic (Tg) rats were quantified using real-time RT-PCR. Results are normalized to bacterial 16S expression (Mean+SD, n = 4–5 rats/group, *p<0.05).
(TIF)

Table S1 **Upregulated** ***B. theta*** **genes in cecal bacteria from Tg vs. nTg Rats.**
(DOCX)

Table S2 **Downregulated** ***B. theta*** **genes in cecal bacteria from Tg vs. nTg Rats.**
(DOCX)

Table S3 **Sequences of oligonucleotides used in PCR reactions.**
(DOCX)
