# Supplementary figures and images for: The Colitis-Associated Transcriptional Profile of Commensal Bacteroides thetaiotaomicron Enhances Adaptive Immune Responses to a Bacterial Antigen
Source: PLoS One. 2012 Aug 3;7(8):e42645. doi: 10.1371/journal.pone.0042645 (PMC3411805; doi:10.1371/journal.pone.0042645)

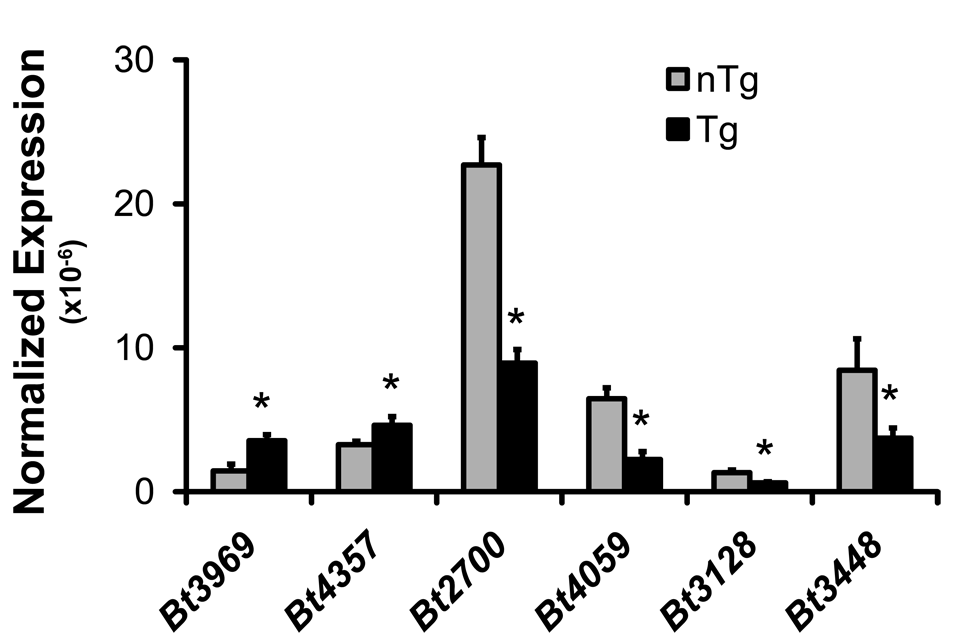

Supplement: Figure S1 — Real-Time RT-PCR Verification of Microarray Results for Selected B. theta Genes. Selected differentially expressed B. theta genes from cecal bacteria from 6 week-monoassociated non-transgenic (nTg) and HLA-B27 transgenic (Tg) rats were quantified using real-time RT-PCR. Results are normalized to bacterial 16S expression (Mean+SD, n = 4–5 rats/group, *p<0.05). (TIF) [file pone.0042645.s001.tif]
