# Supplementary material for: The Colitis-Associated Transcriptional Profile of Commensal Bacteroides thetaiotaomicron Enhances Adaptive Immune Responses to a Bacterial Antigen
Source: PLoS One. 2012 Aug 3;7(8):e42645. doi: 10.1371/journal.pone.0042645 (PMC3411805; doi:10.1371/journal.pone.0042645)
Supplement: Table S1 — Upregulated B. theta genes in cecal bacteria from Tg vs. nTg Rats. (DOCX) [file pone.0042645.s002.docx]

| **Gene Name** | **Fold Change** | **q-value(%)** | **Description** |
| --- | --- | --- | --- |
| BT2259 | 4.18838292 | 2.896784375 | putative lipoprotein |
| BT2260 | 3.614739946 | 2.896784375 | outer membrane protein Omp121 |
| [BT3969](http://genome-www4.stanford.edu/cgi-bin/SMD/source/sourceResult?choice=Gene&option=Name&criteria=BT3969) | 3.20943819 | 0 | cation efflux system protein, AcrB/AcrD/AcrF family protein |
| [BT3968](http://genome-www4.stanford.edu/cgi-bin/SMD/source/sourceResult?choice=Gene&option=Name&criteria=BT3968) | 3.022089442 | 1.309467581 | cation efflux system protein |
| [BT4357](http://genome-www4.stanford.edu/cgi-bin/SMD/source/sourceResult?choice=Gene&option=Name&criteria=BT4357) | 2.33826485 | 0 | SusC homolog |
| [BT3628](http://genome-www4.stanford.edu/cgi-bin/SMD/source/sourceResult?choice=Gene&option=Name&criteria=BT3628) | 2.290907602 | 2.896784375 | PepSY-associated TM helix |
| [BT2896](http://genome-www4.stanford.edu/cgi-bin/SMD/source/sourceResult?choice=Gene&option=Name&criteria=BT2896) | 2.221633128 | 1.309467581 | NHL repeat protein |
| [BT4358](http://genome-www4.stanford.edu/cgi-bin/SMD/source/sourceResult?choice=Gene&option=Name&criteria=BT4358) | 2.194715929 | 0 | SusD homolog |
| [BT2895](http://genome-www4.stanford.edu/cgi-bin/SMD/source/sourceResult?choice=Gene&option=Name&criteria=BT2895) | 2.078715746 | 1.309467581 | endo-1,4-beta-xylanase |
| [BT2894](http://genome-www4.stanford.edu/cgi-bin/SMD/source/sourceResult?choice=Gene&option=Name&criteria=BT2894) | 2.073310185 | 1.309467581 | SusC homolog |
| [BT1416](http://genome-www4.stanford.edu/cgi-bin/SMD/source/sourceResult?choice=Gene&option=Name&criteria=BT1416) | 1.975864849 | 1.309467581 | putative transmembrane protein |
| [BT1417](http://genome-www4.stanford.edu/cgi-bin/SMD/source/sourceResult?choice=Gene&option=Name&criteria=BT1417) | 1.924925776 | 1.309467581 | cytochrome C552 precursor |
| [BT1043](http://genome-www4.stanford.edu/cgi-bin/SMD/source/sourceResult?choice=Gene&option=Name&criteria=BT1043) | 1.893487868 | 2.050224789 | SusD homolog |
| [BT1606](http://genome-www4.stanford.edu/cgi-bin/SMD/source/sourceResult?choice=Gene&option=Name&criteria=BT1606) | 1.871248667 | 1.309467581 | cytochrome C peroxidase |
| [BT2893](http://genome-www4.stanford.edu/cgi-bin/SMD/source/sourceResult?choice=Gene&option=Name&criteria=BT2893) | 1.857155406 | 0 | SusD homolog |
| [BT1044](http://genome-www4.stanford.edu/cgi-bin/SMD/source/sourceResult?choice=Gene&option=Name&criteria=BT1044) | 1.84726727 | 2.050224789 | putative secreted endoglycosidase , GH family 18 |
| [BT1042](http://genome-www4.stanford.edu/cgi-bin/SMD/source/sourceResult?choice=Gene&option=Name&criteria=BT1042) | 1.842180404 | 2.050224789 | SusC homolog |
| [BT3753](http://genome-www4.stanford.edu/cgi-bin/SMD/source/sourceResult?choice=Gene&option=Name&criteria=BT3753) | 1.829649703 | 2.896784375 | endo-beta-N-acetylglucosaminidase F2 precursor (mannosyl-glycoprotein endo-beta-N-acetyl-glucosaminidase F2) |
| [BT3604](http://genome-www4.stanford.edu/cgi-bin/SMD/source/sourceResult?choice=Gene&option=Name&criteria=BT3604) | 1.825184005 | 3.134213019 | SusC homolog |
| [BT1414](http://genome-www4.stanford.edu/cgi-bin/SMD/source/sourceResult?choice=Gene&option=Name&criteria=BT1414) | 1.780158981 | 3.134213019 | conserved hypothetical protein |
| [BT0318](http://genome-www4.stanford.edu/cgi-bin/SMD/source/sourceResult?choice=Gene&option=Name&criteria=BT0318) | 1.77812866 | 0 | SusD homolog |
| [BT2825](http://genome-www4.stanford.edu/cgi-bin/SMD/source/sourceResult?choice=Gene&option=Name&criteria=BT2825) | 1.768322108 | 2.050224789 | chitinase |
| [BT1415](http://genome-www4.stanford.edu/cgi-bin/SMD/source/sourceResult?choice=Gene&option=Name&criteria=BT1415) | 1.761501717 | 5.166828827 | cytochrome c biogenesis protein ccsA |
| [BT2892](http://genome-www4.stanford.edu/cgi-bin/SMD/source/sourceResult?choice=Gene&option=Name&criteria=BT2892) | 1.744863455 | 0 | glycoside hydrolase family 93 |
| [BT0439](http://genome-www4.stanford.edu/cgi-bin/SMD/source/sourceResult?choice=Gene&option=Name&criteria=BT0439) | 1.730794515 | 3.134213019 | SusC homolog |
| [BT4356](http://genome-www4.stanford.edu/cgi-bin/SMD/source/sourceResult?choice=Gene&option=Name&criteria=BT4356) | 1.696663978 | 5.166828827 | putative anti-sigma factor |
| [BT3754](http://genome-www4.stanford.edu/cgi-bin/SMD/source/sourceResult?choice=Gene&option=Name&criteria=BT3754) | 1.678963627 | 2.050224789 | Concanavalin A-like lectin/glucanase |
| [BT0317](http://genome-www4.stanford.edu/cgi-bin/SMD/source/sourceResult?choice=Gene&option=Name&criteria=BT0317) | 1.667438462 | 0 | SusC homolog |
| [BT3752](http://genome-www4.stanford.edu/cgi-bin/SMD/source/sourceResult?choice=Gene&option=Name&criteria=BT3752) | 1.665862767 | 5.166828827 | SusD homolog |
| [BT3751](http://genome-www4.stanford.edu/cgi-bin/SMD/source/sourceResult?choice=Gene&option=Name&criteria=BT3751) | 1.638853626 | 3.134213019 | SusC homolog |
| [BT0027](http://genome-www4.stanford.edu/cgi-bin/SMD/source/sourceResult?choice=Gene&option=Name&criteria=BT0027) | 1.635287443 | 3.134213019 | putative transposase |
| [BT3037](http://genome-www4.stanford.edu/cgi-bin/SMD/source/sourceResult?choice=Gene&option=Name&criteria=BT3037) | 1.635228687 | 2.050224789 | RNA polymerase ECF-type sigma factor |
| [BT3777](http://genome-www4.stanford.edu/cgi-bin/SMD/source/sourceResult?choice=Gene&option=Name&criteria=BT3777) | 1.623709346 | 3.134213019 | hypothetical protein |
| [BT0440](http://genome-www4.stanford.edu/cgi-bin/SMD/source/sourceResult?choice=Gene&option=Name&criteria=BT0440) | 1.622140168 | 3.134213019 | SusD homolog |
| [BT3967](http://genome-www4.stanford.edu/cgi-bin/SMD/source/sourceResult?choice=Gene&option=Name&criteria=BT3967) | 1.6166948 | 2.475294585 | putative two-component system sensor protein histidine kinase |
| [BT1980](http://genome-www4.stanford.edu/cgi-bin/SMD/source/sourceResult?choice=Gene&option=Name&criteria=BT1980) | 1.613481759 | 2.050224789 | transposase |
| [BT2619](http://genome-www4.stanford.edu/cgi-bin/SMD/source/sourceResult?choice=Gene&option=Name&criteria=BT2619) | 1.606694188 | 4.053113941 | two-component system sensor histidine kinase |
| [BT4384](http://genome-www4.stanford.edu/cgi-bin/SMD/source/sourceResult?choice=Gene&option=Name&criteria=BT4384) | 1.598338298 | 4.053113941 | conserved hypothetical protein |
| [BT0054](http://genome-www4.stanford.edu/cgi-bin/SMD/source/sourceResult?choice=Gene&option=Name&criteria=BT0054) | 1.590524553 | 2.050224789 | glycoside transferase family 4 |
| [BT1127](http://genome-www4.stanford.edu/cgi-bin/SMD/source/sourceResult?choice=Gene&option=Name&criteria=BT1127) | 1.582363093 | 1.309467581 | mobilization protein BmgB |
| [BT2622](http://genome-www4.stanford.edu/cgi-bin/SMD/source/sourceResult?choice=Gene&option=Name&criteria=BT2622) | 1.580616515 | 4.053113941 | alpha-glucuronidase |
| [BT1966](http://genome-www4.stanford.edu/cgi-bin/SMD/source/sourceResult?choice=Gene&option=Name&criteria=BT1966) | 1.568668624 | 2.050224789 | multidrug resistance protein, AcrB/AcrD family |
| [BT3674](http://genome-www4.stanford.edu/cgi-bin/SMD/source/sourceResult?choice=Gene&option=Name&criteria=BT3674) | 1.560966509 | 1.309467581 | Six-hairpin glycosidase |
| [BT4681](http://genome-www4.stanford.edu/cgi-bin/SMD/source/sourceResult?choice=Gene&option=Name&criteria=BT4681) | 1.551431769 | 1.309467581 | beta-hexosaminidase precursor |
| [BT3750](http://genome-www4.stanford.edu/cgi-bin/SMD/source/sourceResult?choice=Gene&option=Name&criteria=BT3750) | 1.55000219 | 2.050224789 | SusC homolog |
| [BT1045](http://genome-www4.stanford.edu/cgi-bin/SMD/source/sourceResult?choice=Gene&option=Name&criteria=BT1045) | 1.540779139 | 2.364316466 | Concanavalin A-like lectin/glucanase |
| [BT4779](http://genome-www4.stanford.edu/cgi-bin/SMD/source/sourceResult?choice=Gene&option=Name&criteria=BT4779) | 1.53558471 | 3.134213019 | conserved protein found in conjugate transposon |
| [BT2063](http://genome-www4.stanford.edu/cgi-bin/SMD/source/sourceResult?choice=Gene&option=Name&criteria=BT2063) | 1.525517516 | 2.050224789 | conserved hypothetical protein, putative iron uptake factor |
| [BT2297](http://genome-www4.stanford.edu/cgi-bin/SMD/source/sourceResult?choice=Gene&option=Name&criteria=BT2297) | 1.519783555 | 2.050224789 |  |
| [BT0796](http://genome-www4.stanford.edu/cgi-bin/SMD/source/sourceResult?choice=Gene&option=Name&criteria=BT0796) | 1.519114999 | 5.166828827 | putative helicase |
| [BT0319](http://genome-www4.stanford.edu/cgi-bin/SMD/source/sourceResult?choice=Gene&option=Name&criteria=BT0319) | 1.518789973 | 1.309467581 | conserved hypothetical protein |
| [BT3886](http://genome-www4.stanford.edu/cgi-bin/SMD/source/sourceResult?choice=Gene&option=Name&criteria=BT3886) | 1.516934663 | 2.050224789 | conserved hypothetical protein |
| [BT0209](http://genome-www4.stanford.edu/cgi-bin/SMD/source/sourceResult?choice=Gene&option=Name&criteria=BT0209) | 1.514846428 | 3.134213019 | hypothetical protein |
| [BT4404](http://genome-www4.stanford.edu/cgi-bin/SMD/source/sourceResult?choice=Gene&option=Name&criteria=BT4404) | 1.509555982 | 2.896784375 | SusC homolog |
| [BT1064](http://genome-www4.stanford.edu/cgi-bin/SMD/source/sourceResult?choice=Gene&option=Name&criteria=BT1064) | 1.50210355 | 2.896784375 | Rhodopsin-like GPCR superfamily |
| [BT4403](http://genome-www4.stanford.edu/cgi-bin/SMD/source/sourceResult?choice=Gene&option=Name&criteria=BT4403) | 1.500431737 | 3.134213019 | putative anti-sigma factor |
