# Supplementary material for: The Colitis-Associated Transcriptional Profile of Commensal Bacteroides thetaiotaomicron Enhances Adaptive Immune Responses to a Bacterial Antigen
Source: PLoS One. 2012 Aug 3;7(8):e42645. doi: 10.1371/journal.pone.0042645 (PMC3411805; doi:10.1371/journal.pone.0042645)
Supplement: Table S2 — Downregulated B. theta genes in cecal bacteria from Tg vs. nTg Rats. (DOCX) [file pone.0042645.s003.docx]

| **Gene Name** | **Fold Change** | **q-value(%)** | **Description** |
| --- | --- | --- | --- |
| [BT2555](http://genome-www4.stanford.edu/cgi-bin/SMD/source/sourceResult?choice=Gene&option=Name&criteria=BT2555) | 0.175513 | 4.053114 | hypothetical protein |
| [BT2677](http://genome-www4.stanford.edu/cgi-bin/SMD/source/sourceResult?choice=Gene&option=Name&criteria=BT2677) | 0.193594 | 2.364316 | hypothetical protein |
| [BT4491](http://genome-www4.stanford.edu/cgi-bin/SMD/source/sourceResult?choice=Gene&option=Name&criteria=BT4491) | 0.328931 | 4.053114 | hypothetical protein |
| [BT0505](http://genome-www4.stanford.edu/cgi-bin/SMD/source/sourceResult?choice=Gene&option=Name&criteria=BT0505) | 0.348377 | 2.475295 | Transcription factor, MADS-box |
| [BT4490](http://genome-www4.stanford.edu/cgi-bin/SMD/source/sourceResult?choice=Gene&option=Name&criteria=BT4490) | 0.357235 | 4.053114 | hypothetical protein |
| [BT2962](http://genome-www4.stanford.edu/cgi-bin/SMD/source/sourceResult?choice=Gene&option=Name&criteria=BT2962) | 0.376774 | 2.364316 | hypothetical protein |
| [BT0470](http://genome-www4.stanford.edu/cgi-bin/SMD/source/sourceResult?choice=Gene&option=Name&criteria=BT0470) | 0.389602 | 2.896784 | hypothetical protein , CPS 2 |
| [BT4542](http://genome-www4.stanford.edu/cgi-bin/SMD/source/sourceResult?choice=Gene&option=Name&criteria=BT4542) | 0.390825 | 2.364316 | Type I restriction enzyme EcoR124II specificity protein |
| [BT0490](http://genome-www4.stanford.edu/cgi-bin/SMD/source/sourceResult?choice=Gene&option=Name&criteria=BT0490) | 0.39715 | 2.364316 | conserved hypothetical protein |
| [BT0479](http://genome-www4.stanford.edu/cgi-bin/SMD/source/sourceResult?choice=Gene&option=Name&criteria=BT0479) | 0.398155 | 2.896784 | glycoside transferase family 2, CPS 2 |
| [BT3658](http://genome-www4.stanford.edu/cgi-bin/SMD/source/sourceResult?choice=Gene&option=Name&criteria=BT3658) | 0.399055 | 3.134213 | hypothetical protein |
| [BT0471](http://genome-www4.stanford.edu/cgi-bin/SMD/source/sourceResult?choice=Gene&option=Name&criteria=BT0471) | 0.39936 | 2.475295 | glycoside transferase family 4, CPS 2 |
| [BT1232](http://genome-www4.stanford.edu/cgi-bin/SMD/source/sourceResult?choice=Gene&option=Name&criteria=BT1232) | 0.399855 | 2.364316 | hypothetical protein |
| [BT0172](http://genome-www4.stanford.edu/cgi-bin/SMD/source/sourceResult?choice=Gene&option=Name&criteria=BT0172) | 0.403304 | 2.220402 | hypothetical protein |
| [BT1206](http://genome-www4.stanford.edu/cgi-bin/SMD/source/sourceResult?choice=Gene&option=Name&criteria=BT1206) | 0.406623 | 2.475295 | conserved hypothetical protein |
| [BT2088](http://genome-www4.stanford.edu/cgi-bin/SMD/source/sourceResult?choice=Gene&option=Name&criteria=BT2088) | 0.409643 | 2.364316 |  |
| [BT1318](http://genome-www4.stanford.edu/cgi-bin/SMD/source/sourceResult?choice=Gene&option=Name&criteria=BT1318) | 0.412506 | 3.134213 | signaling protein without kinase domain |
| [BT0294](http://genome-www4.stanford.edu/cgi-bin/SMD/source/sourceResult?choice=Gene&option=Name&criteria=BT0294) | 0.415786 | 2.220402 | Carboxypeptidase regulatory region |
| [BT0462](http://genome-www4.stanford.edu/cgi-bin/SMD/source/sourceResult?choice=Gene&option=Name&criteria=BT0462) | 0.417888 | 2.475295 | putative transcriptional regulator , CPS 2 |
| [BT0081](http://genome-www4.stanford.edu/cgi-bin/SMD/source/sourceResult?choice=Gene&option=Name&criteria=BT0081) | 0.424188 | 2.364316 | conserved hypothetical protein |
| [BT0463](http://genome-www4.stanford.edu/cgi-bin/SMD/source/sourceResult?choice=Gene&option=Name&criteria=BT0463) | 0.430695 | 2.896784 | glucose-1-phosphate thymidylyltransferase , CPS 2 |
| [BT1319](http://genome-www4.stanford.edu/cgi-bin/SMD/source/sourceResult?choice=Gene&option=Name&criteria=BT1319) | 0.431284 | 3.134213 | two-component system response regulator |
| [BT0503](http://genome-www4.stanford.edu/cgi-bin/SMD/source/sourceResult?choice=Gene&option=Name&criteria=BT0503) | 0.442158 | 2.896784 | conserved hypothetical protein |
| [BT1111](http://genome-www4.stanford.edu/cgi-bin/SMD/source/sourceResult?choice=Gene&option=Name&criteria=BT1111) | 0.456027 | 2.220402 |  |
| [BT4560](http://genome-www4.stanford.edu/cgi-bin/SMD/source/sourceResult?choice=Gene&option=Name&criteria=BT4560) | 0.458105 | 3.134213 | thiamine biosynthesis lipoprotein apbE precursor |
| [BT2266](http://genome-www4.stanford.edu/cgi-bin/SMD/source/sourceResult?choice=Gene&option=Name&criteria=BT2266) | 0.459617 | 2.475295 | hypothetical protein |
| [BT2575](http://genome-www4.stanford.edu/cgi-bin/SMD/source/sourceResult?choice=Gene&option=Name&criteria=BT2575) | 0.460118 | 3.134213 | hypothetical protein |
| [BT0476](http://genome-www4.stanford.edu/cgi-bin/SMD/source/sourceResult?choice=Gene&option=Name&criteria=BT0476) | 0.460138 | 2.896784 | D-mannose-1-phosphate guanyltransferase , CPS 2 |
| [BT0469](http://genome-www4.stanford.edu/cgi-bin/SMD/source/sourceResult?choice=Gene&option=Name&criteria=BT0469) | 0.460828 | 4.053114 | hypothetical protein , CPS 2 |
| [BT3743](http://genome-www4.stanford.edu/cgi-bin/SMD/source/sourceResult?choice=Gene&option=Name&criteria=BT3743) | 0.461143 | 3.134213 | conserved hypothetical protein |
| [BT0639](http://genome-www4.stanford.edu/cgi-bin/SMD/source/sourceResult?choice=Gene&option=Name&criteria=BT0639) | 0.462736 | 2.475295 | conserved hypothetical protein |
| [BT2471](http://genome-www4.stanford.edu/cgi-bin/SMD/source/sourceResult?choice=Gene&option=Name&criteria=BT2471) | 0.463319 | 2.475295 | putative outer membrane protein |
| [BT0467](http://genome-www4.stanford.edu/cgi-bin/SMD/source/sourceResult?choice=Gene&option=Name&criteria=BT0467) | 0.466261 | 3.134213 | putative flippase, CPS 2 |
| [BT3942](http://genome-www4.stanford.edu/cgi-bin/SMD/source/sourceResult?choice=Gene&option=Name&criteria=BT3942) | 0.475018 | 2.220402 | Thioesterase superfamily |
| [BT2700](http://genome-www4.stanford.edu/cgi-bin/SMD/source/sourceResult?choice=Gene&option=Name&criteria=BT2700) | 0.47921 | 2.220402 | 50S ribosomal protein L17 |
| [BT0466](http://genome-www4.stanford.edu/cgi-bin/SMD/source/sourceResult?choice=Gene&option=Name&criteria=BT0466) | 0.480061 | 3.134213 | dTDP-glucose 4,6-dehydratase , CPS 2 |
| [BT1508](http://genome-www4.stanford.edu/cgi-bin/SMD/source/sourceResult?choice=Gene&option=Name&criteria=BT1508) | 0.482261 | 2.220402 |  |
| [BT0501](http://genome-www4.stanford.edu/cgi-bin/SMD/source/sourceResult?choice=Gene&option=Name&criteria=BT0501) | 0.486589 | 3.134213 | conserved hypothetical protein |
| [BT4071](http://genome-www4.stanford.edu/cgi-bin/SMD/source/sourceResult?choice=Gene&option=Name&criteria=BT4071) | 0.491722 | 2.220402 | hypothetical protein |
| [BT2669](http://genome-www4.stanford.edu/cgi-bin/SMD/source/sourceResult?choice=Gene&option=Name&criteria=BT2669) | 0.491924 | 2.220402 | conserved hypothetical protein |
| [BT3373](http://genome-www4.stanford.edu/cgi-bin/SMD/source/sourceResult?choice=Gene&option=Name&criteria=BT3373) | 0.495918 | 2.896784 | spermidine n1-acetyltransferase |
| [BT0903](http://genome-www4.stanford.edu/cgi-bin/SMD/source/sourceResult?choice=Gene&option=Name&criteria=BT0903) | 0.497937 | 2.364316 | BatE, TRP domain containing protein |
| [BT2357](http://genome-www4.stanford.edu/cgi-bin/SMD/source/sourceResult?choice=Gene&option=Name&criteria=BT2357) | 0.500174 | 2.896784 | conserved hypothetical protein |
| [BT0171](http://genome-www4.stanford.edu/cgi-bin/SMD/source/sourceResult?choice=Gene&option=Name&criteria=BT0171) | 0.500323 | 2.220402 | Peptidase M |
| [BT0485](http://genome-www4.stanford.edu/cgi-bin/SMD/source/sourceResult?choice=Gene&option=Name&criteria=BT0485) | 0.500931 | 2.475295 |  |
| [BT4158](http://genome-www4.stanford.edu/cgi-bin/SMD/source/sourceResult?choice=Gene&option=Name&criteria=BT4158) | 0.501184 | 2.364316 | Lipolytic enzyme, G-D-S-L |
| [BT0874](http://genome-www4.stanford.edu/cgi-bin/SMD/source/sourceResult?choice=Gene&option=Name&criteria=BT0874) | 0.50341 | 3.134213 | GtrA-like protein |
| [BT4413](http://genome-www4.stanford.edu/cgi-bin/SMD/source/sourceResult?choice=Gene&option=Name&criteria=BT4413) | 0.50428 | 2.220402 | conserved hypothetical protein |
| [BT0857](http://genome-www4.stanford.edu/cgi-bin/SMD/source/sourceResult?choice=Gene&option=Name&criteria=BT0857) | 0.504349 | 2.220402 | outer membrane protein TolC, putative |
| [BT0468](http://genome-www4.stanford.edu/cgi-bin/SMD/source/sourceResult?choice=Gene&option=Name&criteria=BT0468) | 0.504627 | 4.053114 | putative F420H2-dehydrogenase 40 kDa subunit , CPS 2 |
| [BT2487](http://genome-www4.stanford.edu/cgi-bin/SMD/source/sourceResult?choice=Gene&option=Name&criteria=BT2487) | 0.509965 | 2.220402 |  |
| [BT3466](http://genome-www4.stanford.edu/cgi-bin/SMD/source/sourceResult?choice=Gene&option=Name&criteria=BT3466) | 0.512054 | 2.220402 | hypothetical protein |
| [BT2422](http://genome-www4.stanford.edu/cgi-bin/SMD/source/sourceResult?choice=Gene&option=Name&criteria=BT2422) | 0.513038 | 2.364316 | conserved hypothetical protein |
| [BT0464](http://genome-www4.stanford.edu/cgi-bin/SMD/source/sourceResult?choice=Gene&option=Name&criteria=BT0464) | 0.513804 | 4.053114 | dTDP-4-dehydrorhamnose 3,5-epimerase , CPS 2 |
| [BT0160](http://genome-www4.stanford.edu/cgi-bin/SMD/source/sourceResult?choice=Gene&option=Name&criteria=BT0160) | 0.517556 | 2.220402 | conserved hypothetical protein |
| [BT2676](http://genome-www4.stanford.edu/cgi-bin/SMD/source/sourceResult?choice=Gene&option=Name&criteria=BT2676) | 0.518572 | 2.364316 | conserved hypothetical protein |
| [BT2833](http://genome-www4.stanford.edu/cgi-bin/SMD/source/sourceResult?choice=Gene&option=Name&criteria=BT2833) | 0.520547 | 2.364316 | similar to endonuclease/exonuclease/phosphatase |
| [BT1748](http://genome-www4.stanford.edu/cgi-bin/SMD/source/sourceResult?choice=Gene&option=Name&criteria=BT1748) | 0.521605 | 2.220402 | hypothetical protein |
| [BT4283](http://genome-www4.stanford.edu/cgi-bin/SMD/source/sourceResult?choice=Gene&option=Name&criteria=BT4283) | 0.524921 | 4.053114 | conserved hypothetical protein |
| [BT0772](http://genome-www4.stanford.edu/cgi-bin/SMD/source/sourceResult?choice=Gene&option=Name&criteria=BT0772) | 0.525496 | 2.220402 | putative lipoprotein |
| [BT1914](http://genome-www4.stanford.edu/cgi-bin/SMD/source/sourceResult?choice=Gene&option=Name&criteria=BT1914) | 0.528952 | 2.220402 | thioredoxin-like protein, putative thioredoxin |
| [BT0966](http://genome-www4.stanford.edu/cgi-bin/SMD/source/sourceResult?choice=Gene&option=Name&criteria=BT0966) | 0.534544 | 2.364316 | RNA polymerase ECF-type sigma factor |
| [BT4532](http://genome-www4.stanford.edu/cgi-bin/SMD/source/sourceResult?choice=Gene&option=Name&criteria=BT4532) | 0.538856 | 4.053114 | hypothetical protein |
| [BT4640](http://genome-www4.stanford.edu/cgi-bin/SMD/source/sourceResult?choice=Gene&option=Name&criteria=BT4640) | 0.538974 | 1.41859 | conserved hypothetical protein |
| [BT0499](http://genome-www4.stanford.edu/cgi-bin/SMD/source/sourceResult?choice=Gene&option=Name&criteria=BT0499) | 0.539022 | 4.053114 | cation efflux system protein |
| [BT3619](http://genome-www4.stanford.edu/cgi-bin/SMD/source/sourceResult?choice=Gene&option=Name&criteria=BT3619) | 0.541901 | 2.364316 | putative transmembrane protein |
| [BT4068](http://genome-www4.stanford.edu/cgi-bin/SMD/source/sourceResult?choice=Gene&option=Name&criteria=BT4068) | 0.54278 | 2.364316 | conserved hypothetical protein |
| [BT2356](http://genome-www4.stanford.edu/cgi-bin/SMD/source/sourceResult?choice=Gene&option=Name&criteria=BT2356) | 0.542923 | 3.134213 | transcriptional regulator, AraC family |
| [BT3995](http://genome-www4.stanford.edu/cgi-bin/SMD/source/sourceResult?choice=Gene&option=Name&criteria=BT3995) | 0.543891 | 2.364316 | alanyl-tRNA synthetase |
| [BT2074](http://genome-www4.stanford.edu/cgi-bin/SMD/source/sourceResult?choice=Gene&option=Name&criteria=BT2074) | 0.549583 | 2.896784 | ketol-acid reductoisomerase |
| [BT0043](http://genome-www4.stanford.edu/cgi-bin/SMD/source/sourceResult?choice=Gene&option=Name&criteria=BT0043) | 0.550379 | 3.134213 | putative glycosyltransferase |
| [BT0762](http://genome-www4.stanford.edu/cgi-bin/SMD/source/sourceResult?choice=Gene&option=Name&criteria=BT0762) | 0.55143 | 2.896784 | hypothetical protein |
| [BT1392](http://genome-www4.stanford.edu/cgi-bin/SMD/source/sourceResult?choice=Gene&option=Name&criteria=BT1392) | 0.552264 | 2.220402 | conserved hypothetical protein |
| [BT2675](http://genome-www4.stanford.edu/cgi-bin/SMD/source/sourceResult?choice=Gene&option=Name&criteria=BT2675) | 0.553262 | 2.364316 | hypothetical protein |
| [BT4059](http://genome-www4.stanford.edu/cgi-bin/SMD/source/sourceResult?choice=Gene&option=Name&criteria=BT4059) | 0.553414 | 2.220402 | NADH dehydrogenase I, chain M |
| [BT4070](http://genome-www4.stanford.edu/cgi-bin/SMD/source/sourceResult?choice=Gene&option=Name&criteria=BT4070) | 0.555535 | 2.475295 | RNA polymerase ECF-type sigma factor |
| [BT0473](http://genome-www4.stanford.edu/cgi-bin/SMD/source/sourceResult?choice=Gene&option=Name&criteria=BT0473) | 0.555577 | 4.053114 | glycoside transferase family 2, CPS 2 |
| [BT3210](http://genome-www4.stanford.edu/cgi-bin/SMD/source/sourceResult?choice=Gene&option=Name&criteria=BT3210) | 0.557454 | 2.220402 | cell division protein FtsX |
| [BT4067](http://genome-www4.stanford.edu/cgi-bin/SMD/source/sourceResult?choice=Gene&option=Name&criteria=BT4067) | 0.557702 | 2.220402 | NADH dehydrogenase I, chain A |
| [BT3430](http://genome-www4.stanford.edu/cgi-bin/SMD/source/sourceResult?choice=Gene&option=Name&criteria=BT3430) | 0.557921 | 2.364316 | ribosomal protein S20 |
| [BT3582](http://genome-www4.stanford.edu/cgi-bin/SMD/source/sourceResult?choice=Gene&option=Name&criteria=BT3582) | 0.55829 | 2.475295 | hypothetical protein |
| [BT1428](http://genome-www4.stanford.edu/cgi-bin/SMD/source/sourceResult?choice=Gene&option=Name&criteria=BT1428) | 0.558862 | 2.364316 | Antibiotic biosynthesis monooxyg |
| [BT1472](http://genome-www4.stanford.edu/cgi-bin/SMD/source/sourceResult?choice=Gene&option=Name&criteria=BT1472) | 0.55888 | 2.220402 | glycoside transferase family 2 |
| [BT4646](http://genome-www4.stanford.edu/cgi-bin/SMD/source/sourceResult?choice=Gene&option=Name&criteria=BT4646) | 0.560224 | 2.475295 | conserved hypothetical protein |
| [BT3539](http://genome-www4.stanford.edu/cgi-bin/SMD/source/sourceResult?choice=Gene&option=Name&criteria=BT3539) | 0.56126 | 2.220402 | hypothetical protein |
| [BT1462](http://genome-www4.stanford.edu/cgi-bin/SMD/source/sourceResult?choice=Gene&option=Name&criteria=BT1462) | 0.56259 | 3.134213 | conserved hypothetical protein |
| [BT0585](http://genome-www4.stanford.edu/cgi-bin/SMD/source/sourceResult?choice=Gene&option=Name&criteria=BT0585) | 0.563199 | 1.41859 | Flavin reductase-like, FMN-binding |
| [BT3691](http://genome-www4.stanford.edu/cgi-bin/SMD/source/sourceResult?choice=Gene&option=Name&criteria=BT3691) | 0.565056 | 0 | conserved hypothetical protein, putative membrane protein |
| [BT3690](http://genome-www4.stanford.edu/cgi-bin/SMD/source/sourceResult?choice=Gene&option=Name&criteria=BT3690) | 0.565334 | 2.364316 | putative membrane protein |
| [BT4197](http://genome-www4.stanford.edu/cgi-bin/SMD/source/sourceResult?choice=Gene&option=Name&criteria=BT4197) | 0.568225 | 4.053114 | hypothetical protein |
| [BT4478](http://genome-www4.stanford.edu/cgi-bin/SMD/source/sourceResult?choice=Gene&option=Name&criteria=BT4478) | 0.568806 | 2.364316 | hypothetical protein |
| [BT3212](http://genome-www4.stanford.edu/cgi-bin/SMD/source/sourceResult?choice=Gene&option=Name&criteria=BT3212) | 0.570456 | 3.134213 | putative bacitracin resistance protein |
| [BT4142](http://genome-www4.stanford.edu/cgi-bin/SMD/source/sourceResult?choice=Gene&option=Name&criteria=BT4142) | 0.571076 | 2.220402 | possible AraC family transcriptional regulator |
| [BT2463](http://genome-www4.stanford.edu/cgi-bin/SMD/source/sourceResult?choice=Gene&option=Name&criteria=BT2463) | 0.571324 | 2.364316 | RNA polymerase ECF-type sigma factor |
| [BT2168](http://genome-www4.stanford.edu/cgi-bin/SMD/source/sourceResult?choice=Gene&option=Name&criteria=BT2168) | 0.575856 | 2.364316 | coproporphyrinogen III oxidase |
| [BT2661](http://genome-www4.stanford.edu/cgi-bin/SMD/source/sourceResult?choice=Gene&option=Name&criteria=BT2661) | 0.575959 | 2.896784 | hypothetical protein |
| [BT1471](http://genome-www4.stanford.edu/cgi-bin/SMD/source/sourceResult?choice=Gene&option=Name&criteria=BT1471) | 0.575966 | 2.220402 | putative transmembrane protein |
| [BT2365](http://genome-www4.stanford.edu/cgi-bin/SMD/source/sourceResult?choice=Gene&option=Name&criteria=BT2365) | 0.576451 | 2.220402 | SusD homolog |
| [BT4257](http://genome-www4.stanford.edu/cgi-bin/SMD/source/sourceResult?choice=Gene&option=Name&criteria=BT4257) | 0.577256 | 2.475295 | putative histidinol-phosphatase |
| [BT4315](http://genome-www4.stanford.edu/cgi-bin/SMD/source/sourceResult?choice=Gene&option=Name&criteria=BT4315) | 0.578279 | 4.053114 | HPr(Ser) phosphatase |
| [BT1424](http://genome-www4.stanford.edu/cgi-bin/SMD/source/sourceResult?choice=Gene&option=Name&criteria=BT1424) | 0.579075 | 2.475295 | Histone-like bacterial DNA-binding |
| [BT2633](http://genome-www4.stanford.edu/cgi-bin/SMD/source/sourceResult?choice=Gene&option=Name&criteria=BT2633) | 0.579732 | 2.364316 | hypothetical protein |
| [BT3747](http://genome-www4.stanford.edu/cgi-bin/SMD/source/sourceResult?choice=Gene&option=Name&criteria=BT3747) | 0.580304 | 2.896784 | conserved hypothetical protein |
| [BT0828](http://genome-www4.stanford.edu/cgi-bin/SMD/source/sourceResult?choice=Gene&option=Name&criteria=BT0828) | 0.581039 | 2.220402 | dTDP-4-dehydrorhamnose 3,5-epimerase |
| [BT1207](http://genome-www4.stanford.edu/cgi-bin/SMD/source/sourceResult?choice=Gene&option=Name&criteria=BT1207) | 0.581118 | 2.364316 | glycerate dehydrogenase (NADH-dependent) |
| [BT1005](http://genome-www4.stanford.edu/cgi-bin/SMD/source/sourceResult?choice=Gene&option=Name&criteria=BT1005) | 0.581157 | 2.896784 | hypothetical protein |
| [BT4757](http://genome-www4.stanford.edu/cgi-bin/SMD/source/sourceResult?choice=Gene&option=Name&criteria=BT4757) | 0.582442 | 2.896784 | hypothetical protein |
| [BT2223](http://genome-www4.stanford.edu/cgi-bin/SMD/source/sourceResult?choice=Gene&option=Name&criteria=BT2223) | 0.583922 | 3.134213 | TPR-repeat-containing protein |
| [BT1670](http://genome-www4.stanford.edu/cgi-bin/SMD/source/sourceResult?choice=Gene&option=Name&criteria=BT1670) | 0.584478 | 2.220402 | putative transmembrane transport protein |
| [BT0947](http://genome-www4.stanford.edu/cgi-bin/SMD/source/sourceResult?choice=Gene&option=Name&criteria=BT0947) | 0.585053 | 2.220402 | integrase |
| [BT0991](http://genome-www4.stanford.edu/cgi-bin/SMD/source/sourceResult?choice=Gene&option=Name&criteria=BT0991) | 0.585176 | 1.41859 | putative transporter |
| [BT4540](http://genome-www4.stanford.edu/cgi-bin/SMD/source/sourceResult?choice=Gene&option=Name&criteria=BT4540) | 0.587292 | 2.220402 | putative Type I restriction enzyme MjaXP specificity protein |
| [BT2680](http://genome-www4.stanford.edu/cgi-bin/SMD/source/sourceResult?choice=Gene&option=Name&criteria=BT2680) | 0.587716 | 2.364316 | beta-galactosidase |
| [BT1671](http://genome-www4.stanford.edu/cgi-bin/SMD/source/sourceResult?choice=Gene&option=Name&criteria=BT1671) | 0.589566 | 0 | endonuclease III |
| [BT0805](http://genome-www4.stanford.edu/cgi-bin/SMD/source/sourceResult?choice=Gene&option=Name&criteria=BT0805) | 0.590359 | 2.220402 | putative membrane protein |
| [BT4591](http://genome-www4.stanford.edu/cgi-bin/SMD/source/sourceResult?choice=Gene&option=Name&criteria=BT4591) | 0.590627 | 2.364316 | putative nitrogen utilization substance protein |
| [BT3213](http://genome-www4.stanford.edu/cgi-bin/SMD/source/sourceResult?choice=Gene&option=Name&criteria=BT3213) | 0.590846 | 4.053114 | tRNA pseudouridine synthase B |
| [BT3577](http://genome-www4.stanford.edu/cgi-bin/SMD/source/sourceResult?choice=Gene&option=Name&criteria=BT3577) | 0.59156 | 4.053114 | putative protease |
| [BT2685](http://genome-www4.stanford.edu/cgi-bin/SMD/source/sourceResult?choice=Gene&option=Name&criteria=BT2685) | 0.591973 | 2.220402 | conserved hypothetical protein |
| [BT2938](http://genome-www4.stanford.edu/cgi-bin/SMD/source/sourceResult?choice=Gene&option=Name&criteria=BT2938) | 0.593167 | 3.134213 | glycoside transferase family 4 |
| [BT4151](http://genome-www4.stanford.edu/cgi-bin/SMD/source/sourceResult?choice=Gene&option=Name&criteria=BT4151) | 0.593638 | 2.220402 | beta-glucuronidase |
| [BT3211](http://genome-www4.stanford.edu/cgi-bin/SMD/source/sourceResult?choice=Gene&option=Name&criteria=BT3211) | 0.594074 | 3.134213 | conserved hypothetical protein |
| [BT1027](http://genome-www4.stanford.edu/cgi-bin/SMD/source/sourceResult?choice=Gene&option=Name&criteria=BT1027) | 0.594446 | 3.134213 | hypothetical protein |
| [BT1208](http://genome-www4.stanford.edu/cgi-bin/SMD/source/sourceResult?choice=Gene&option=Name&criteria=BT1208) | 0.594746 | 2.896784 | Cellular Component: integral to membrane (GO:1621) |
| [BT4351](http://genome-www4.stanford.edu/cgi-bin/SMD/source/sourceResult?choice=Gene&option=Name&criteria=BT4351) | 0.595213 | 2.220402 | Peptidase M, neutral zinc metallopeptidases, zinc-binding site |
| [BT1388](http://genome-www4.stanford.edu/cgi-bin/SMD/source/sourceResult?choice=Gene&option=Name&criteria=BT1388) | 0.596481 | 2.364316 | integral membrane protein |
| [BT1964](http://genome-www4.stanford.edu/cgi-bin/SMD/source/sourceResult?choice=Gene&option=Name&criteria=BT1964) | 0.596586 | 4.053114 | Bacterial regulatory protein, Te |
| [BT0181](http://genome-www4.stanford.edu/cgi-bin/SMD/source/sourceResult?choice=Gene&option=Name&criteria=BT0181) | 0.596746 | 2.220402 | putative NADH dehydrogenase/NAD(P)H nitroreductase |
| [BT4186](http://genome-www4.stanford.edu/cgi-bin/SMD/source/sourceResult?choice=Gene&option=Name&criteria=BT4186) | 0.597999 | 2.220402 | conserved hypothetical protein |
| [BT1531](http://genome-www4.stanford.edu/cgi-bin/SMD/source/sourceResult?choice=Gene&option=Name&criteria=BT1531) | 0.598439 | 2.220402 | two-component system sensor histidine kinase |
| [BT3763](http://genome-www4.stanford.edu/cgi-bin/SMD/source/sourceResult?choice=Gene&option=Name&criteria=BT3763) | 0.598446 | 2.220402 | rhamnulose kinase/L-fuculose kinase |
| [BT3815](http://genome-www4.stanford.edu/cgi-bin/SMD/source/sourceResult?choice=Gene&option=Name&criteria=BT3815) | 0.598533 | 3.134213 | putative membrane protein |
| [BT4058](http://genome-www4.stanford.edu/cgi-bin/SMD/source/sourceResult?choice=Gene&option=Name&criteria=BT4058) | 0.598782 | 2.220402 | NADH dehydrogenase I, chain N |
| [BT3653](http://genome-www4.stanford.edu/cgi-bin/SMD/source/sourceResult?choice=Gene&option=Name&criteria=BT3653) | 0.599474 | 2.220402 | hypothetical protein |
| [BT0666](http://genome-www4.stanford.edu/cgi-bin/SMD/source/sourceResult?choice=Gene&option=Name&criteria=BT0666) | 0.600708 | 2.364316 | hypothetical protein |
| [BT4214](http://genome-www4.stanford.edu/cgi-bin/SMD/source/sourceResult?choice=Gene&option=Name&criteria=BT4214) | 0.600848 | 2.364316 | hydrolase of the alpha/beta superfamily |
| [BT3748](http://genome-www4.stanford.edu/cgi-bin/SMD/source/sourceResult?choice=Gene&option=Name&criteria=BT3748) | 0.601501 | 2.364316 | RNA polymerase ECF-type sigma factor |
| [BT0628](http://genome-www4.stanford.edu/cgi-bin/SMD/source/sourceResult?choice=Gene&option=Name&criteria=BT0628) | 0.601766 | 2.364316 | conserved hypothetical protein |
| [BT1693](http://genome-www4.stanford.edu/cgi-bin/SMD/source/sourceResult?choice=Gene&option=Name&criteria=BT1693) | 0.602147 | 3.134213 | periplasmic linker protein, putative multidrug resistance protein |
| [BT3018](http://genome-www4.stanford.edu/cgi-bin/SMD/source/sourceResult?choice=Gene&option=Name&criteria=BT3018) | 0.602167 | 1.41859 | putative mechano-sensitive ion channel |
| [BT1949](http://genome-www4.stanford.edu/cgi-bin/SMD/source/sourceResult?choice=Gene&option=Name&criteria=BT1949) | 0.60282 | 2.896784 | conserved hypothetical protein |
| [BT4635](http://genome-www4.stanford.edu/cgi-bin/SMD/source/sourceResult?choice=Gene&option=Name&criteria=BT4635) | 0.603365 | 2.220402 | putative anti-sigma factor |
| [BT0724](http://genome-www4.stanford.edu/cgi-bin/SMD/source/sourceResult?choice=Gene&option=Name&criteria=BT0724) | 0.605352 | 2.364316 | hypothetical protein |
| [BT1278](http://genome-www4.stanford.edu/cgi-bin/SMD/source/sourceResult?choice=Gene&option=Name&criteria=BT1278) | 0.605439 | 2.364316 | RNA polymerase ECF-type sigma factor |
| [BT4561](http://genome-www4.stanford.edu/cgi-bin/SMD/source/sourceResult?choice=Gene&option=Name&criteria=BT4561) | 0.606442 | 3.134213 | conserved hypothetical protein, putative integral membrane protein |
| [BT1162](http://genome-www4.stanford.edu/cgi-bin/SMD/source/sourceResult?choice=Gene&option=Name&criteria=BT1162) | 0.606457 | 3.134213 | putative outer membrane protein |
| [BT0953](http://genome-www4.stanford.edu/cgi-bin/SMD/source/sourceResult?choice=Gene&option=Name&criteria=BT0953) | 0.606842 | 2.475295 | integrase |
| [BT1867](http://genome-www4.stanford.edu/cgi-bin/SMD/source/sourceResult?choice=Gene&option=Name&criteria=BT1867) | 0.606847 | 2.364316 | hypothetical protein |
| [BT0584](http://genome-www4.stanford.edu/cgi-bin/SMD/source/sourceResult?choice=Gene&option=Name&criteria=BT0584) | 0.607733 | 2.364316 | putative outer membrane protein |
| [BT1698](http://genome-www4.stanford.edu/cgi-bin/SMD/source/sourceResult?choice=Gene&option=Name&criteria=BT1698) | 0.609093 | 2.364316 | putative oxaloacetate decarboxylase gamma chain |
| [BT2267](http://genome-www4.stanford.edu/cgi-bin/SMD/source/sourceResult?choice=Gene&option=Name&criteria=BT2267) | 0.609235 | 2.220402 | integrase protein |
| [BT1439](http://genome-www4.stanford.edu/cgi-bin/SMD/source/sourceResult?choice=Gene&option=Name&criteria=BT1439) | 0.609545 | 2.364316 | SusD homolog |
| [BT1850](http://genome-www4.stanford.edu/cgi-bin/SMD/source/sourceResult?choice=Gene&option=Name&criteria=BT1850) | 0.61039 | 2.220402 | putative transmembrane acyltransferase protein |
| [BT3989](http://genome-www4.stanford.edu/cgi-bin/SMD/source/sourceResult?choice=Gene&option=Name&criteria=BT3989) | 0.610571 | 2.364316 | Ankyrin |
| [BT4180](http://genome-www4.stanford.edu/cgi-bin/SMD/source/sourceResult?choice=Gene&option=Name&criteria=BT4180) | 0.610755 | 3.134213 | acetyl xylan esterase A |
| [BT1907](http://genome-www4.stanford.edu/cgi-bin/SMD/source/sourceResult?choice=Gene&option=Name&criteria=BT1907) | 0.611283 | 2.364316 | putative RNA polymerase sigma factor RpoS |
| [BT3128](http://genome-www4.stanford.edu/cgi-bin/SMD/source/sourceResult?choice=Gene&option=Name&criteria=BT3128) | 0.61133 | 2.896784 | putative xanthosine triphosphate pyrophosphatase |
| [BT3688](http://genome-www4.stanford.edu/cgi-bin/SMD/source/sourceResult?choice=Gene&option=Name&criteria=BT3688) | 0.612163 | 2.220402 | putative acetyltransferase |
| [BT3532](http://genome-www4.stanford.edu/cgi-bin/SMD/source/sourceResult?choice=Gene&option=Name&criteria=BT3532) | 0.612264 | 1.41859 | aldose 1-epimerase precursor |
| [BT3209](http://genome-www4.stanford.edu/cgi-bin/SMD/source/sourceResult?choice=Gene&option=Name&criteria=BT3209) | 0.612673 | 2.364316 | similar to SAM dependent methyltransferase |
| [BT4574](http://genome-www4.stanford.edu/cgi-bin/SMD/source/sourceResult?choice=Gene&option=Name&criteria=BT4574) | 0.612743 | 2.220402 | Camphor resistance CrcB protein |
| [BT4533](http://genome-www4.stanford.edu/cgi-bin/SMD/source/sourceResult?choice=Gene&option=Name&criteria=BT4533) | 0.612748 | 4.053114 | Prefoldin |
| [BT2161](http://genome-www4.stanford.edu/cgi-bin/SMD/source/sourceResult?choice=Gene&option=Name&criteria=BT2161) | 0.613147 | 2.896784 | 50S ribosomal protein L9 |
| [BT3735](http://genome-www4.stanford.edu/cgi-bin/SMD/source/sourceResult?choice=Gene&option=Name&criteria=BT3735) | 0.613788 | 2.220402 | hypothetical protein |
| [BT3730](http://genome-www4.stanford.edu/cgi-bin/SMD/source/sourceResult?choice=Gene&option=Name&criteria=BT3730) | 0.615278 | 2.364316 | putative regulatory protein |
| [BT1564](http://genome-www4.stanford.edu/cgi-bin/SMD/source/sourceResult?choice=Gene&option=Name&criteria=BT1564) | 0.615857 | 2.896784 | putative GTP-cyclohydrolase protein |
| [BT0175](http://genome-www4.stanford.edu/cgi-bin/SMD/source/sourceResult?choice=Gene&option=Name&criteria=BT0175) | 0.61608 | 2.896784 | conserved hypothetical protein |
| [BT2330](http://genome-www4.stanford.edu/cgi-bin/SMD/source/sourceResult?choice=Gene&option=Name&criteria=BT2330) | 0.617711 | 3.134213 | putative Toll-Interleukin receptor |
| [BT0569](http://genome-www4.stanford.edu/cgi-bin/SMD/source/sourceResult?choice=Gene&option=Name&criteria=BT0569) | 0.617916 | 4.053114 | putative acylhydrolase |
| [BT2248](http://genome-www4.stanford.edu/cgi-bin/SMD/source/sourceResult?choice=Gene&option=Name&criteria=BT2248) | 0.617981 | 2.220402 | putative integral membrane protein |
| [BT3742](http://genome-www4.stanford.edu/cgi-bin/SMD/source/sourceResult?choice=Gene&option=Name&criteria=BT3742) | 0.618712 | 2.364316 | conserved hypothetical protein |
| [BT1478](http://genome-www4.stanford.edu/cgi-bin/SMD/source/sourceResult?choice=Gene&option=Name&criteria=BT1478) | 0.618932 | 2.364316 | putative histidinol-phosphatase |
| [BT2430](http://genome-www4.stanford.edu/cgi-bin/SMD/source/sourceResult?choice=Gene&option=Name&criteria=BT2430) | 0.619484 | 2.220402 | hexokinase type III |
| [BT2591](http://genome-www4.stanford.edu/cgi-bin/SMD/source/sourceResult?choice=Gene&option=Name&criteria=BT2591) | 0.620051 | 2.364316 | hypothetical protein |
| [BT2046](http://genome-www4.stanford.edu/cgi-bin/SMD/source/sourceResult?choice=Gene&option=Name&criteria=BT2046) | 0.620753 | 3.134213 | putative cardiolipin synthetase |
| [BT2470](http://genome-www4.stanford.edu/cgi-bin/SMD/source/sourceResult?choice=Gene&option=Name&criteria=BT2470) | 0.621908 | 2.364316 | putative outer membrane protein |
| [BT4589](http://genome-www4.stanford.edu/cgi-bin/SMD/source/sourceResult?choice=Gene&option=Name&criteria=BT4589) | 0.622312 | 2.364316 | putative 50S ribosomal protein L25 |
| [BT3618](http://genome-www4.stanford.edu/cgi-bin/SMD/source/sourceResult?choice=Gene&option=Name&criteria=BT3618) | 0.622713 | 3.134213 | conserved hypothetical protein |
| [BT0744](http://genome-www4.stanford.edu/cgi-bin/SMD/source/sourceResult?choice=Gene&option=Name&criteria=BT0744) | 0.623101 | 2.364316 | 2-amino-4-hydroxy-6- hydroxymethyldihydropteridine pyrophosphokinase |
| [BT3651](http://genome-www4.stanford.edu/cgi-bin/SMD/source/sourceResult?choice=Gene&option=Name&criteria=BT3651) | 0.623213 | 2.220402 | conserved hypothetical protein |
| [BT4687](http://genome-www4.stanford.edu/cgi-bin/SMD/source/sourceResult?choice=Gene&option=Name&criteria=BT4687) | 0.623713 | 3.134213 | Helix-hairpin-helix motif, class |
| [BT3692](http://genome-www4.stanford.edu/cgi-bin/SMD/source/sourceResult?choice=Gene&option=Name&criteria=BT3692) | 0.623731 | 2.364316 | phosphate acetyltransferase |
| [BT0713](http://genome-www4.stanford.edu/cgi-bin/SMD/source/sourceResult?choice=Gene&option=Name&criteria=BT0713) | 0.623787 | 0 | conserved hypothetical protein |
| [BT1999](http://genome-www4.stanford.edu/cgi-bin/SMD/source/sourceResult?choice=Gene&option=Name&criteria=BT1999) | 0.6239 | 2.364316 | anaerobic ribonucleoside-triphosphate reductase activating protein |
| [BT1726](http://genome-www4.stanford.edu/cgi-bin/SMD/source/sourceResult?choice=Gene&option=Name&criteria=BT1726) | 0.625047 | 2.364316 | integrase , CPS 6 |
| [BT1271](http://genome-www4.stanford.edu/cgi-bin/SMD/source/sourceResult?choice=Gene&option=Name&criteria=BT1271) | 0.625237 | 2.220402 | 30S ribosomal protein S16 |
| [BT0640](http://genome-www4.stanford.edu/cgi-bin/SMD/source/sourceResult?choice=Gene&option=Name&criteria=BT0640) | 0.626167 | 2.475295 | Fe-S oxidoreductases family 2 |
| [BT1799](http://genome-www4.stanford.edu/cgi-bin/SMD/source/sourceResult?choice=Gene&option=Name&criteria=BT1799) | 0.628398 | 2.220402 | putative vitamin B12 receptor precursor |
| [BT0956](http://genome-www4.stanford.edu/cgi-bin/SMD/source/sourceResult?choice=Gene&option=Name&criteria=BT0956) | 0.628446 | 2.220402 | ABC-2 type transporter |
| [BT2359](http://genome-www4.stanford.edu/cgi-bin/SMD/source/sourceResult?choice=Gene&option=Name&criteria=BT2359) | 0.629032 | 2.475295 | formamidopyrimidine-DNA glycosylase |
| [BT2413](http://genome-www4.stanford.edu/cgi-bin/SMD/source/sourceResult?choice=Gene&option=Name&criteria=BT2413) | 0.629161 | 2.364316 | homoserine O-succinyltransferase |
| [BT3760](http://genome-www4.stanford.edu/cgi-bin/SMD/source/sourceResult?choice=Gene&option=Name&criteria=BT3760) | 0.630031 | 2.896784 | argininosuccinate synthase |
| [BT4590](http://genome-www4.stanford.edu/cgi-bin/SMD/source/sourceResult?choice=Gene&option=Name&criteria=BT4590) | 0.631627 | 3.134213 | conserved hypothetical protein |
| [BT3248](http://genome-www4.stanford.edu/cgi-bin/SMD/source/sourceResult?choice=Gene&option=Name&criteria=BT3248) | 0.632188 | 2.364316 | putative metal ABC transporter, ATP-binding protein |
| [BT0342](http://genome-www4.stanford.edu/cgi-bin/SMD/source/sourceResult?choice=Gene&option=Name&criteria=BT0342) | 0.632871 | 3.134213 | putative 5-methyltetrahydrofolate-homocystein m... |
| [BT3273](http://genome-www4.stanford.edu/cgi-bin/SMD/source/sourceResult?choice=Gene&option=Name&criteria=BT3273) | 0.634049 | 2.220402 | hypothetical protein |
| [BT0566](http://genome-www4.stanford.edu/cgi-bin/SMD/source/sourceResult?choice=Gene&option=Name&criteria=BT0566) | 0.634446 | 2.220402 | alanyl-tRNA synthetase |
| [BT2779](http://genome-www4.stanford.edu/cgi-bin/SMD/source/sourceResult?choice=Gene&option=Name&criteria=BT2779) | 0.634967 | 2.364316 | 3-demethylubiquinone-9 3-methyltransferase |
| [BT3371](http://genome-www4.stanford.edu/cgi-bin/SMD/source/sourceResult?choice=Gene&option=Name&criteria=BT3371) | 0.635783 | 2.364316 | glycoside transferase family 2 |
| [BT3818](http://genome-www4.stanford.edu/cgi-bin/SMD/source/sourceResult?choice=Gene&option=Name&criteria=BT3818) | 0.637109 | 2.475295 | conserved hypothetical protein |
| [BT4325](http://genome-www4.stanford.edu/cgi-bin/SMD/source/sourceResult?choice=Gene&option=Name&criteria=BT4325) | 0.637264 | 2.475295 | similar to efflux ABC transporter, permease protein |
| [BT4183](http://genome-www4.stanford.edu/cgi-bin/SMD/source/sourceResult?choice=Gene&option=Name&criteria=BT4183) | 0.637669 | 2.896784 | pectate lyase L precursor |
| [BT0926](http://genome-www4.stanford.edu/cgi-bin/SMD/source/sourceResult?choice=Gene&option=Name&criteria=BT0926) | 0.638058 | 2.364316 | ABC-type transport, permease protein |
| [BT0292](http://genome-www4.stanford.edu/cgi-bin/SMD/source/sourceResult?choice=Gene&option=Name&criteria=BT0292) | 0.638161 | 2.475295 | conserved hypothetical protein |
| [BT4390](http://genome-www4.stanford.edu/cgi-bin/SMD/source/sourceResult?choice=Gene&option=Name&criteria=BT4390) | 0.640126 | 4.053114 | B3/4 |
| [BT3584](http://genome-www4.stanford.edu/cgi-bin/SMD/source/sourceResult?choice=Gene&option=Name&criteria=BT3584) | 0.64056 | 2.475295 | thiamine biosynthesis lipoprotein ApbE |
| [BT0829](http://genome-www4.stanford.edu/cgi-bin/SMD/source/sourceResult?choice=Gene&option=Name&criteria=BT0829) | 0.640698 | 2.475295 | UDP-glucose 6-dehydrogenase |
| [BT1830](http://genome-www4.stanford.edu/cgi-bin/SMD/source/sourceResult?choice=Gene&option=Name&criteria=BT1830) | 0.641107 | 4.053114 | 10 kDa chaperonin (groES) |
| [BT3219](http://genome-www4.stanford.edu/cgi-bin/SMD/source/sourceResult?choice=Gene&option=Name&criteria=BT3219) | 0.641481 | 2.364316 | S-adenosylmethionine synthetase |
| [BT2668](http://genome-www4.stanford.edu/cgi-bin/SMD/source/sourceResult?choice=Gene&option=Name&criteria=BT2668) | 0.641905 | 2.896784 | MotA/TolQ/ExbB proton channel |
| [BT3357](http://genome-www4.stanford.edu/cgi-bin/SMD/source/sourceResult?choice=Gene&option=Name&criteria=BT3357) | 0.64314 | 2.220402 | ribonuclease III |
| [BT1205](http://genome-www4.stanford.edu/cgi-bin/SMD/source/sourceResult?choice=Gene&option=Name&criteria=BT1205) | 0.643195 | 2.475295 | putative ATPase, AAA family |
| [BT2001](http://genome-www4.stanford.edu/cgi-bin/SMD/source/sourceResult?choice=Gene&option=Name&criteria=BT2001) | 0.644232 | 2.896784 | membrane-associated zinc metalloprotease |
| [BT0727](http://genome-www4.stanford.edu/cgi-bin/SMD/source/sourceResult?choice=Gene&option=Name&criteria=BT0727) | 0.644289 | 2.364316 | clostripain-related protein |
| [BT3378](http://genome-www4.stanford.edu/cgi-bin/SMD/source/sourceResult?choice=Gene&option=Name&criteria=BT3378) | 0.644426 | 2.220402 | GtrA-like protein |
| [BT2412](http://genome-www4.stanford.edu/cgi-bin/SMD/source/sourceResult?choice=Gene&option=Name&criteria=BT2412) | 0.644582 | 2.475295 | putative collagenase |
| [BT3933](http://genome-www4.stanford.edu/cgi-bin/SMD/source/sourceResult?choice=Gene&option=Name&criteria=BT3933) | 0.645502 | 2.475295 | chorismate mutase/prephenate dehydratase (tyrA) |
| [BT0786](http://genome-www4.stanford.edu/cgi-bin/SMD/source/sourceResult?choice=Gene&option=Name&criteria=BT0786) | 0.646297 | 2.475295 | putative integral membrane protein |
| [BT1829](http://genome-www4.stanford.edu/cgi-bin/SMD/source/sourceResult?choice=Gene&option=Name&criteria=BT1829) | 0.646737 | 3.134213 | 60 kDa chaperonin (groEL) |
| [BT4638](http://genome-www4.stanford.edu/cgi-bin/SMD/source/sourceResult?choice=Gene&option=Name&criteria=BT4638) | 0.647692 | 4.053114 | conserved hypothetical protein |
| [BT3276](http://genome-www4.stanford.edu/cgi-bin/SMD/source/sourceResult?choice=Gene&option=Name&criteria=BT3276) | 0.647756 | 2.220402 | conserved hypothetical protein |
| [BT2342](http://genome-www4.stanford.edu/cgi-bin/SMD/source/sourceResult?choice=Gene&option=Name&criteria=BT2342) | 0.647764 | 2.364316 | transcription regulator |
| [BT4489](http://genome-www4.stanford.edu/cgi-bin/SMD/source/sourceResult?choice=Gene&option=Name&criteria=BT4489) | 0.648166 | 3.134213 | conserved hypothetical protein |
| [BT0841](http://genome-www4.stanford.edu/cgi-bin/SMD/source/sourceResult?choice=Gene&option=Name&criteria=BT0841) | 0.648659 | 2.475295 | hypothetical protein |
| [BT2211](http://genome-www4.stanford.edu/cgi-bin/SMD/source/sourceResult?choice=Gene&option=Name&criteria=BT2211) | 0.648856 | 3.134213 | putative transmembrane protein |
| [BT3578](http://genome-www4.stanford.edu/cgi-bin/SMD/source/sourceResult?choice=Gene&option=Name&criteria=BT3578) | 0.650152 | 2.364316 | conserved hypothetical protein |
| [BT4764](http://genome-www4.stanford.edu/cgi-bin/SMD/source/sourceResult?choice=Gene&option=Name&criteria=BT4764) | 0.650479 | 2.364316 |  |
| [BT1677](http://genome-www4.stanford.edu/cgi-bin/SMD/source/sourceResult?choice=Gene&option=Name&criteria=BT1677) | 0.650507 | 2.220402 | putative hydrolase |
| [BT3478](http://genome-www4.stanford.edu/cgi-bin/SMD/source/sourceResult?choice=Gene&option=Name&criteria=BT3478) | 0.650924 | 3.134213 | integrase |
| [BT4366](http://genome-www4.stanford.edu/cgi-bin/SMD/source/sourceResult?choice=Gene&option=Name&criteria=BT4366) | 0.65103 | 2.364316 | putative transcription regulator |
| [BT1728](http://genome-www4.stanford.edu/cgi-bin/SMD/source/sourceResult?choice=Gene&option=Name&criteria=BT1728) | 0.651076 | 2.220402 | RNA polymerase ECF-type sigma factor |
| [BT4066](http://genome-www4.stanford.edu/cgi-bin/SMD/source/sourceResult?choice=Gene&option=Name&criteria=BT4066) | 0.651441 | 2.364316 | NADH dehydrogenase I, chain B |
| [BT2957](http://genome-www4.stanford.edu/cgi-bin/SMD/source/sourceResult?choice=Gene&option=Name&criteria=BT2957) | 0.652488 | 2.220402 | transcriptional regulator |
| [BT4665](http://genome-www4.stanford.edu/cgi-bin/SMD/source/sourceResult?choice=Gene&option=Name&criteria=BT4665) | 0.652535 | 2.364316 | K+ uptake protein |
| [BT2069](http://genome-www4.stanford.edu/cgi-bin/SMD/source/sourceResult?choice=Gene&option=Name&criteria=BT2069) | 0.652589 | 2.475295 | GCN5-related N-acetyltransferase |
| [BT4096](http://genome-www4.stanford.edu/cgi-bin/SMD/source/sourceResult?choice=Gene&option=Name&criteria=BT4096) | 0.652716 | 0 | lipolytic enzyme, G-D-S-L |
| [BT1252](http://genome-www4.stanford.edu/cgi-bin/SMD/source/sourceResult?choice=Gene&option=Name&criteria=BT1252) | 0.654226 | 2.364316 | possible transmembrane protein |
| [BT1855](http://genome-www4.stanford.edu/cgi-bin/SMD/source/sourceResult?choice=Gene&option=Name&criteria=BT1855) | 0.65535 | 2.364316 | putative methyl transferase |
| [BT3283](http://genome-www4.stanford.edu/cgi-bin/SMD/source/sourceResult?choice=Gene&option=Name&criteria=BT3283) | 0.65577 | 2.896784 | conserved hypothetical protein with conserved domain |
| [BT3656](http://genome-www4.stanford.edu/cgi-bin/SMD/source/sourceResult?choice=Gene&option=Name&criteria=BT3656) | 0.655926 | 4.053114 | putative beta-xylosidase |
| [BT2525](http://genome-www4.stanford.edu/cgi-bin/SMD/source/sourceResult?choice=Gene&option=Name&criteria=BT2525) | 0.656469 | 2.475295 | cephalosporin-C deacetylase |
| [BT2102](http://genome-www4.stanford.edu/cgi-bin/SMD/source/sourceResult?choice=Gene&option=Name&criteria=BT2102) | 0.656771 | 2.220402 | putative cAMP-binding domain, regulatory protein |
| [BT4636](http://genome-www4.stanford.edu/cgi-bin/SMD/source/sourceResult?choice=Gene&option=Name&criteria=BT4636) | 0.657031 | 2.364316 | RNA polymerase ECF-type sigma factor |
| [BT3745](http://genome-www4.stanford.edu/cgi-bin/SMD/source/sourceResult?choice=Gene&option=Name&criteria=BT3745) | 0.657085 | 2.896784 | conserved hypothetical protein |
| [BT0746](http://genome-www4.stanford.edu/cgi-bin/SMD/source/sourceResult?choice=Gene&option=Name&criteria=BT0746) | 0.657214 | 3.134213 | putative zinc protease |
| [BT0677](http://genome-www4.stanford.edu/cgi-bin/SMD/source/sourceResult?choice=Gene&option=Name&criteria=BT0677) | 0.657349 | 2.896784 | thioredoxin (TRX) |
| [BT4240](http://genome-www4.stanford.edu/cgi-bin/SMD/source/sourceResult?choice=Gene&option=Name&criteria=BT4240) | 0.658275 | 2.364316 | conserved hypothetical protein, with a phosphotransferase enzyme family domain |
| [BT3583](http://genome-www4.stanford.edu/cgi-bin/SMD/source/sourceResult?choice=Gene&option=Name&criteria=BT3583) | 0.659369 | 2.896784 | putative dehydrogenases and relate proteins |
| [BT3726](http://genome-www4.stanford.edu/cgi-bin/SMD/source/sourceResult?choice=Gene&option=Name&criteria=BT3726) | 0.659484 | 2.475295 | undecaprenyl pyrophosphate synthetase |
| [BT1253](http://genome-www4.stanford.edu/cgi-bin/SMD/source/sourceResult?choice=Gene&option=Name&criteria=BT1253) | 0.659853 | 2.220402 | Winged helix repressor DNA-bindi |
| [BT0570](http://genome-www4.stanford.edu/cgi-bin/SMD/source/sourceResult?choice=Gene&option=Name&criteria=BT0570) | 0.660542 | 2.475295 | excinuclease ABC subunit B |
| [BT3074](http://genome-www4.stanford.edu/cgi-bin/SMD/source/sourceResult?choice=Gene&option=Name&criteria=BT3074) | 0.660543 | 2.220402 | Phosphoesterase, PA-phosphatase |
| [BT1915](http://genome-www4.stanford.edu/cgi-bin/SMD/source/sourceResult?choice=Gene&option=Name&criteria=BT1915) | 0.66066 | 2.364316 | pyruvate carboxylase subunit A |
| [BT3274](http://genome-www4.stanford.edu/cgi-bin/SMD/source/sourceResult?choice=Gene&option=Name&criteria=BT3274) | 0.660691 | 2.220402 | hypothetical protein |
| [BT1613](http://genome-www4.stanford.edu/cgi-bin/SMD/source/sourceResult?choice=Gene&option=Name&criteria=BT1613) | 0.660818 | 2.364316 | Outer membrane chaperone Skp (OmpH) |
| [BT0664](http://genome-www4.stanford.edu/cgi-bin/SMD/source/sourceResult?choice=Gene&option=Name&criteria=BT0664) | 0.661371 | 2.896784 | conserved hypothetical protein |
| [BT4698](http://genome-www4.stanford.edu/cgi-bin/SMD/source/sourceResult?choice=Gene&option=Name&criteria=BT4698) | 0.661439 | 2.896784 | conserved hypothetical protein |
| [BT3010](http://genome-www4.stanford.edu/cgi-bin/SMD/source/sourceResult?choice=Gene&option=Name&criteria=BT3010) | 0.661464 | 2.364316 | RNA polymerase ECF-type sigma factor |
| [BT3759](http://genome-www4.stanford.edu/cgi-bin/SMD/source/sourceResult?choice=Gene&option=Name&criteria=BT3759) | 0.661699 | 4.053114 | N-acetyl-gamma-glutamyl-phosphate reductase |
| [BT1862](http://genome-www4.stanford.edu/cgi-bin/SMD/source/sourceResult?choice=Gene&option=Name&criteria=BT1862) | 0.661768 | 2.364316 | hypothetical protein |
| [BT0920](http://genome-www4.stanford.edu/cgi-bin/SMD/source/sourceResult?choice=Gene&option=Name&criteria=BT0920) | 0.662049 | 2.364316 | putative O-sialoglycoprotein endopeptidase |
| [BT0834](http://genome-www4.stanford.edu/cgi-bin/SMD/source/sourceResult?choice=Gene&option=Name&criteria=BT0834) | 0.662049 | 4.053114 | putative permease |
| [BT3744](http://genome-www4.stanford.edu/cgi-bin/SMD/source/sourceResult?choice=Gene&option=Name&criteria=BT3744) | 0.662133 | 2.364316 | conserved hypothetical protein |
| [BT2802](http://genome-www4.stanford.edu/cgi-bin/SMD/source/sourceResult?choice=Gene&option=Name&criteria=BT2802) | 0.662481 | 2.364316 | conserved hypothetical protein |
| [BT3936](http://genome-www4.stanford.edu/cgi-bin/SMD/source/sourceResult?choice=Gene&option=Name&criteria=BT3936) | 0.66272 | 2.364316 | prephenate dehydratase |
| [BT0146](http://genome-www4.stanford.edu/cgi-bin/SMD/source/sourceResult?choice=Gene&option=Name&criteria=BT0146) | 0.662898 | 2.896784 | unsaturated glucuronyl hydrolase |
| [BT4215](http://genome-www4.stanford.edu/cgi-bin/SMD/source/sourceResult?choice=Gene&option=Name&criteria=BT4215) | 0.664799 | 2.475295 | shikimate 5-dehydrogenase |
| [BT3650](http://genome-www4.stanford.edu/cgi-bin/SMD/source/sourceResult?choice=Gene&option=Name&criteria=BT3650) | 0.665115 | 2.220402 | putative lemA protein |
| [BT3250](http://genome-www4.stanford.edu/cgi-bin/SMD/source/sourceResult?choice=Gene&option=Name&criteria=BT3250) | 0.665796 | 4.053114 | conserved hypothetical protein |
| [BT1870](http://genome-www4.stanford.edu/cgi-bin/SMD/source/sourceResult?choice=Gene&option=Name&criteria=BT1870) | 0.665906 | 2.220402 |  |
| [BT4123](http://genome-www4.stanford.edu/cgi-bin/SMD/source/sourceResult?choice=Gene&option=Name&criteria=BT4123) | 0.66656 | 4.053114 | exo-poly-alpha-D-galacturonosidase precursor |
| [BT3448](http://genome-www4.stanford.edu/cgi-bin/SMD/source/sourceResult?choice=Gene&option=Name&criteria=BT3448) | 0.666624 | 2.364316 | UDP-N-acetylglucosamine--N-acetylmuramyl- (pentapeptide) pyrophosphoryl-undecaprenol N-acetylglucosamine transferase |
| [BT3682](http://genome-www4.stanford.edu/cgi-bin/SMD/source/sourceResult?choice=Gene&option=Name&criteria=BT3682) | 0.666634 | 4.053114 | Calcium-binding EF-hand |
