# Supplementary material for: The Colitis-Associated Transcriptional Profile of Commensal Bacteroides thetaiotaomicron Enhances Adaptive Immune Responses to a Bacterial Antigen
Source: PLoS One. 2012 Aug 3;7(8):e42645. doi: 10.1371/journal.pone.0042645 (PMC3411805; doi:10.1371/journal.pone.0042645)
Supplement: Table S3 — Sequences of oligonucleotides used in PCR reactions. (DOCX) [file pone.0042645.s004.docx]

| Primer Name | Sequence (5’to 3’) |
| --- | --- |
| rIFNgF | AACAGTAAAGCAAAAAAGGATGCA |
| rIFNgR | TGCTGGATCTGTGGGTTGTTC |
| rTNFaF | GACCCTCACACTCAGATCATCTTCT |
| rTNFaR | CGCTGGCTCAGCCACTCC |
| rActnF | GCCCTGGCTCCTAGCACC |
| rActnR | CCACCAATCCACACAGAGTACTTG |
| BtFtsKF | TTCCGTGTATCCGCCATGA |
| BtFtsKR | TACCAATCAGGCGGTTTGC |
| BtFtsQF | CGCTGGAACGGGAACTGA |
| BtFtsQR | CGCTGGGTGTTTTGTAGCATT |
| BtFtsXF | TGGGCGGTGACGTACGA |
| BtFtsXR | TGCCGCAGACAATGAGCAT |
| Bt2260F | AACCGGGTGTTATTCTTCAGGAT |
| Bt2260R | TGCTGCCAGTAGTCCTGCATA |
| Bt3969F | TGCTGCGTCCCGTATTCC |
| Bt3969R | CGATTGCCATCGGGACAA |
| Bt4357F | TGCGTTTGCCGGTTGTATATC |
| Bt4357R | TTGGAAGGCGTATTCGTCATC |
| Bt2895F | CATTGGAGTGCTGCGGAGAT |
| Bt2895R | AACCACTCCCTGATCCGAAA |
| Bt2700F | TCTCACAGAAGCGCTATGTTATCTAAC |
| Bt2700R | CCTTTGCCACAGTCGTAGTGATT |
| Bt4059F | TGGTTCCCGCTTACCTTCCT |
| Bt4059R | CCATGTGTGGAACGGGAAA |
| Bt3128F | CCGAAGGGTACGAACAGACTTT |
| Bt3128R | CCAGTGCACGGTGACTGATC |
| Bt3448F | GACCGGACGCGAAAATCTTA |
| Bt3448R | GGGAACCCGCTGCATCT |
| Bt16SF | CAGTGTCAGTTGCAGTCCAGTGA |
| Bt16SR | GTGTAGCGGTGAAATGCTTAGATATC |
